# Preparation of 16β-Estradiol Derivative Libraries as Bisubstrate Inhibitors of 17β-Hydroxysteroid Dehydrogenase Type 1 Using the Multidetachable Sulfamate Linker

**DOI:** 10.3390/molecules15031590

**Published:** 2010-03-10

**Authors:** Marie Bérubé, Florian Delagoutte, Donald Poirier

**Affiliations:** Laboratory of Medicinal Chemistry, CHUQ (CHUL), Research Center and Laval University, Quebec, G1V 4G2, Canada

**Keywords:** Solid-phase synthesis, sulfamate linker, steroid, inhibitor, 17β-HSD

## Abstract

Combinatorial chemistry is a powerful tool used to rapidly generate a large number of potentially biologically active compounds. In our goal to develop bisubstrate inhibitors of 17β-hydroxysteroid dehydrogenase type 1 (17β-HSD1) that interact with both the substrate (estrone or estradiol) and the cofactor (NAD(P)H) binding sites, we used parallel solid-phase synthesis to prepare three libraries of 16β-estradiol derivatives with two or three levels of molecular diversity. From estrone, we first synthesized a sulfamate precursor that we loaded on trityl chloride resin using the efficient multidetachable sulfamate linker strategy recently developed in our laboratory. We then introduced molecular diversity [one or two amino acid(s) followed by a carboxylic acid] on steroid nucleus by Fmoc peptide chemistry. Finally, after a nucleophilic cleavage, libraries of 30, 63 and 25 estradiol derivatives were provided. A library of 30 sulfamoylated estradiol derivatives was also generated by acidic cleavage and its members were screened for inhibition of steroid sulfatase. Biological evaluation on homogenated HEK-293 cells overexpressing 17β-HSD1 of the estradiol derivatives carrying different oligoamide-type chains at C-16 first revealed that three levels of molecular diversity (a spacer of two amino acids) were necessary to interact with the adenosine part of the cofactor binding site. Second, the best inhibition was obtained when hydrophobic residues (phenylalanine) were used as building blocks.

## 1. Introduction

**Figure 1:**
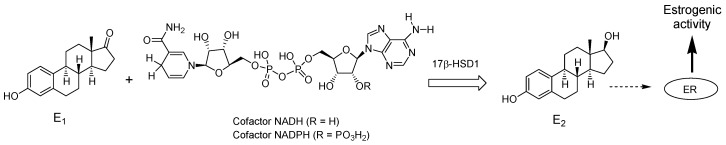
17β-HSD1 uses the cofactor NAD(P)H to transform estrone (E_1_) into estradiol (E_2_), the most potent estrogen.

17β-Hydroxysteroid dehydrogenase type 1 (17β-HSD1), also called human estradiol dehydrogenase [E.C.1.1.1.62], is a protein comprised of 327 amino acids that exists as a homodimer of 35 kDa [1]. This very important steroidogenic enzyme catalyzes the last step in the biosynthesis of estradiol (E_2_), the most potent estrogen [2,3,4]. For this purpose, this enzyme uses the cofactor NAD(P)H to stereoselectively reduce the C17 ketone of estrone (E_1_) into a 17β-alcohol, as illustrated in Figure 1. Because E_2_ concentration has been found to be significantly higher in breast tumours than in normal breast tissue [5,6] 17β-HSD1 is an interesting therapeutic target for estrogen-sensitive diseases such as breast cancer.

**Figure 2:**
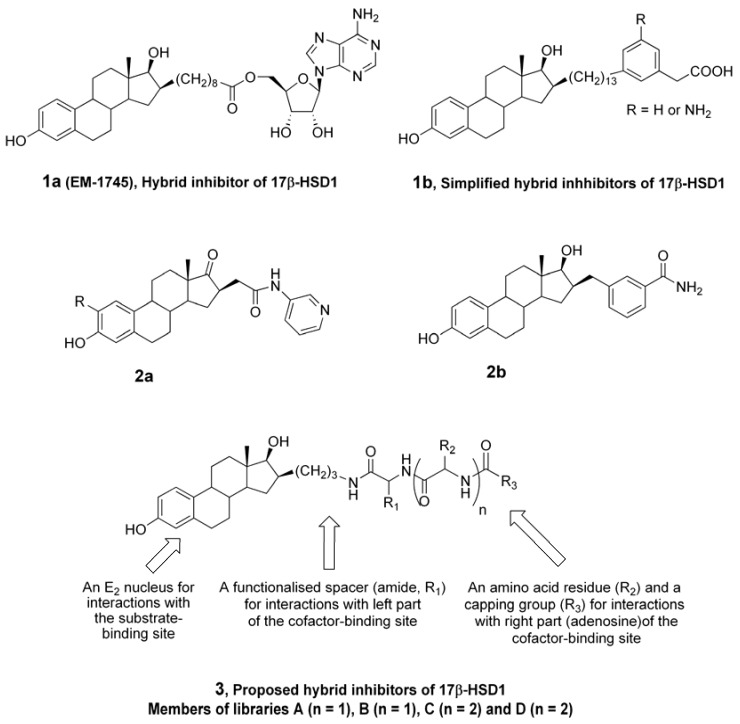
Bisubstrate hybrid inhibitors **1a** and **1b**, simplified inhibitors **2a** and **2b**, and general structure **3** of proposed inhibitors of 17β-HSD1.

Although 17β-HSD1 activity was reported 50 years ago [7,8], no significant progress was made to obtain active inhibitors as drugs [9,10,11,12,13,14]. Among the known inhibitors [9] the bisubstrate category is promising because these inhibitors interact with both the substrate (E_1_) and the cofactor (NAD(P)H) binding sites. In collaboration with Dr. S.X. Lin, our research group developed the first bisubstrate inhibitors of 17β-HSD1 by linking E_2_ and adenosine with an alkyl side-chain spacer. With a spacer of eight methylenes, EM-1745 (**1a**, Figure 2) was found to be the best bisubstrate inhibitor of that series [15,16]. EM-1745 (**1a**) was also co-crystallized with the enzyme and X-ray analysis revealed that **1a** interacts with both the substrate (E_2_) and the cofactor (adenosine part of NADH) enzyme binding sites [17]. The amine and the alcohol groups on the adenosine moiety of **1a** form hydrogen bonds with Asp65 and Ser11, respectively while the steroid nucleus forms hydrogen bonds with His221, Ser142 and Tyr155. Simplified bisubstrate inhibitors were also developed by our research group in order to improve the stability and the bioavailability of **1a** [18,19]. The carboxylic acid derivatives **1b** were found to be the best inhibitors of that series. In order to explain the high potential of compound **1b** as inhibitors of 17β-HSD1, we hypothesized that, as a bioisostere of a phosphate, the carboxylic acid functional group should form hydrogen bonds with one or more of the same amino acids interacting with the phosphate of NADPH (Ser11, Arg37 and Lys195) [19,20,21]. More recently, a new generation of hybrid inhibitors of 17β-HSD1 was reported by Sterix [22,23,24] and by us [25]. Lawrence *et al*. [22,23,24] have reported potent inhibitors of 17β-HSD1 structurally composed of a pyridyl moiety linked by an amide at C16 position of E_1_ (compounds **2a**). Using the GOLD docking program, they determine that the steroid moiety interacts with the substrate-binding site while the pyridyl moiety forms interactions with the nicotinamide part of the cofactor. On the other hand, we reported a new family of 16β-E_2_ derivatives (compounds **2b**) [25] having a *m*-carbamoylbenzyl moiety that interacts with the enzyme as demonstrated by the 3D analysis of the ternary (17β-HSD1/**2b**/NADP) complex [26]. Taken together, the results discussed above indicate that the enzyme contains important amino acids, which could be targeted with an appropriate side-chain added at position 16β of E_2_.

**Figure 3:**
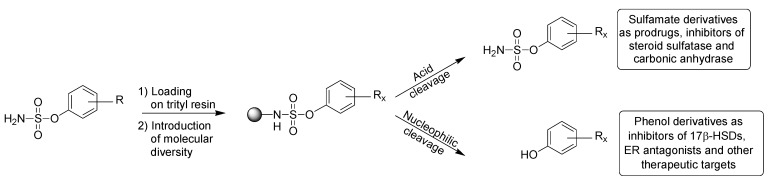
Versatility of the multidetachable sulfamate linker for generating sulfamate and phenol derivatives by acidic or nucleophilic cleavage, respectively.

In order to improve the affinity of the bisubstrate inhibitors **1a** and **1b** for 17β-HSD1, a series of E_2_ derivatives was designed. Thus, the 16β-methylene side-chain of bisubstrate inhibitor **1** was replaced by functional groups that potentially interact with the left part (nicotinamide and spacer) of the cofactor-binding site. On the other hand, an amine and/or a carboxylic acid as functional group to form interactions with the right part (adenosine) of the cofactor-binding site was added at the spacer end. In fact, the amine group on the adenosine moiety of **1a** and the carboxylic acid function on **1b** were found to be important for the interactions with the cofactor-binding site of this enzyme. To rapidly provide a large number of 16β-E_2_ derivatives of general structure **3** for structure activity relationship (SAR) study, parallel solid-phase synthesis was used. Among the resins and the linkers known for preparing libraries of E_2_ derivatives by solid-phase chemistry [27] the linker sulfamate on trityl chloride resin developed by our research group was chosen [28,29]. This multidetachable type linker can generate a phenol or a sulfamate derivative depending on cleavage conditions as presented in Figure 3. It could be used for the synthesis of steroidal and non-steroidal compounds, thus adding to its potential. Furthermore, this linker has already been used to successfully prepare several libraries of E_2_ derivatives, sulfamoylated or not [30,31,32]. The members of these libraries provided 17β-HSD1 inhibitors or steroid sulfatase potential inhibitors, respectively [32]. We report herein the preparation of one library of sulfamoylated E_2_ derivatives (library A: 30 members) and three libraries of E_2_ derivatives (libraries B, C and D: 30, 63 and 25 members, respectively). Biological evaluation of members of libraries B and C on homogenated HEK-293 cells overexpressing 17β-HSD1 will also be presented and discussed. Finally, the chemical problems met during the preparation of library D will be discussed and solutions will be proposed.

## 2. Results and Discussion

New bisubstrate inhibitors of 17β-HSD1 that should interact with the substrate binding site and the left (nicotinamide and spacer) and the right (adenosine) part of the cofactor binding site were designed and prepared by parallel solid-phase synthesis. One or two amino acid building blocks were introduced at position C16β of the E_2_ scaffold linked on solid support by the sulfamate linker, followed by the addition of a carboxylic acid building block. After removal of the protective group and nucleophilic cleavage, peptidosteroids with the general structure **3** were generated. The preparation of peptidosteroids was chosen since side chain of natural and non-natural amino acids provides an interesting pool of functionalities. Furthermore, amino acid coupling procedures are well known for solid-phase synthesis and the Fmoc coupling strategy could be easily used with the sulfamate linker.

### 2.1. Synthesis of steroidal solid-phase precursors **6a** and **6b** in solution (Scheme 1)

**Scheme 1:**
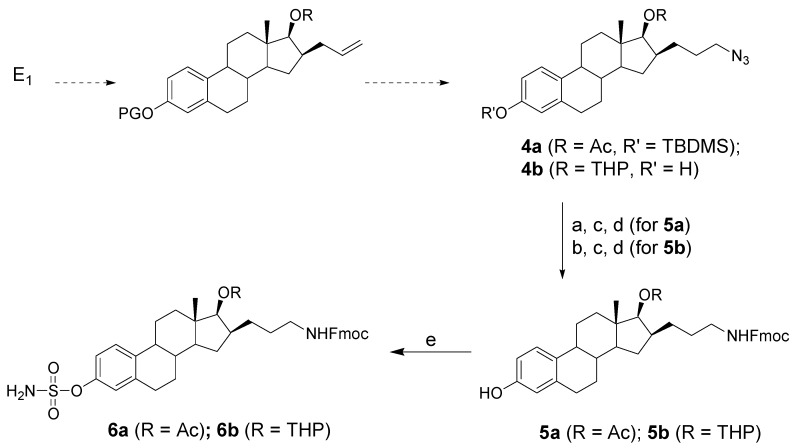
Synthesis of solid-phase precursors **6a** and **6b**.

Before the preparation of libraries A-D, compounds **6a** and **6b** were synthesized in solution as presented in Scheme 1. Azide intermediates **4a** and **4b** were prepared as reported previously in 8 and 9 steps, respectively, from commercially available E_1_ [33]. Since amino acids will be introduced using the Fmoc-coupling procedure, a Fmoc on the aminopropyl side chain at position C16β was selected as an appropriate protective group. For this purpose, azides **4a** and **4b** were first reduced using hydrogen catalyzed by Pd/C. The C3 silylated protective group was next removed using TBAF in THF. Compounds **5a** and **5b** were subsequently obtained by selective protection of the amine with a Fmoc group. Treatment of **5a** and **5b** in DCM with sulfamoyl chloride [34] in the presence of a non-nucleophilic base, 2,6-di-*t*-butyl-4-methylpyridine, provided solid-phase precursors **6a** and **6b** in 4 and 3 steps (82% and 52% overall yield, respectively) from **4**.

### 2.2. Synthesis of carboxylic acid building blocks ***14–18*** (Scheme 2)

For library C members, capping with an amine functional group was chosen to interact with the cofactor (adenosine) binding site. For this purpose, aniline derivatives were chosen. In order to obtain optimal interactions with the cofactor-binding site of the enzyme, carboxylic acid with several alkyl spacer lengths (n = 0 to 3 methylenes) was chosen. Aniline derivatives **9–10** with a spacer of two methylenes were not commercially available, but were prepared easily in one step from 4-aminocinnamic acid or 3-nitrocinnamic acid, respectively, as previously reported [35]. In order to avoid polymerisation during the capping coupling step on solid-phase organic synthesis, free anilines **7–11** were protected as Fmoc using FmocOSu and NaHCO_3_ in a mixture THF/H_2_O (5:1) to provide **14–18** in good yields (51–93%). It is noteworthy to mention that Fmoc-aniline derivatives **12–13** were commercially available.

### 2.3. Solid-phase synthesis of libraries A, B and C (Scheme 3)

A library of 30 sulfamoylated E_2_ derivatives (A), a library of 30 E_2_ derivatives (B), and a library of 63 E_2_ derivatives (C) were prepared by parallel solid-phase synthesis using the multidetachable linker sulfamate. Precursor **6a** was first loaded on trityl chloride resin. For this reaction, trityl chloride resin was swelled in dry DCM and treated with **6a** and diisopropylethylamine (DIPEA) in a peptide flask. After 16 h of shaking, the reaction mixture was filtered and washed with DCM and MeOH to obtain resin **19**. The loading yield of **19** was calculated by the increase of the resin weight. This yield was 70% for libraries A and B and 42% for library C. A lower loading yield was obtained for library C because 1 equivalent of **6a** was used for 2 equivalents of trityl chloride resin instead of 1 equivalent of resin used in the preparation of libraries A and B. On a model library with a loading of 75%, completion of the coupling reaction was very difficult for the introduction of the third level of molecular diversity. It was hypothesized that steric hindrance could be responsible for the lower reactivity of the amine on the steroid. Therefore, less precursor **6a** was loaded on resin when more than two levels of molecular diversity needed to be introduced on the steroid. In the next step, resin **19** was treated for 1 h with a freshly prepared solution of 20% piperidine in DCM to remove the Fmoc protective group and to free the amine for the next step. It is noteworthy to mention that after each solid-phase organic step, the resin was washed with the appropriate solvent and dried under a vacuum. Furthermore, the solid-phase reactions were monitored by a mini-cleavage test of a random sampling of resin with 5% TFA in DCM.

The resin **20** was next split into 30 equal portions for libraries A and B and 63 equal portions for library C. The resins were then placed in bottom fritted reaction vessels of a 96 solid-phase reaction block of an ACT-Labtech semi-automated synthesizer. The first level of molecular diversity (R_i_) was introduced on each resin **20** with one of a selection of Fmoc-protected amino acids from *L* series. Fmoc-*L*-Phe-OH, Fmoc-*L*-Ile-OH, Fmoc-*L*-Pro-OH, Fmoc-*L*-Ala-OH and Fmoc-Gly-OH were used as building blocks for libraries A and B, and Fmoc-*L*-Phe-OH, Fmoc-*L*-4-NO_2_-Phe-OH and Fmoc-Gly-OH were chosen as building blocks for library C. The coupling reaction was performed for 2 to 3 h with DIPEA in DMF using PyBOP and HOBt as coupling reagents. The Fmoc protective group was then removed as described above to afford resins **22** from **21**. Introduction of a second level of molecular diversity (n = 2) on resins **22** using the same procedure and Fmoc-*L*-amino acids (Phe, 4-NO_2_-Phe and Gly) was carried on for library C. Removal of Fmoc group then provided resins **24** from **23**. For members of both libraries, a second or a third level of molecular diversity (R_j_) was introduced using a selection of carboxylic acids as building blocks (4-pyridylacetic acid, benzoic acid, acid **14**, 2-pyrazinecarboxylic acid, 3-aminopyrazine-2-carboxylic acid, and indole-2-carboxylic acid for libraries A and B, and carboxylic acids **12–18** for library C). These carboxylic acids were preactivated with PyBrOP and HOBt. The reaction was carried on for 2 to 3 h with DIPEA in DMF. Then, resins **25** (n = 1 or 2) that were prepared with carboxylic acids **12–18** as building blocks were treated with a solution of 20% piperidine in DCM in order to remove the Fmoc protective group. Finally, the C17-acetate protective group of resins **26** and **27** was removed by treatment with MeONa in MeOH and THF to afford resins **28**.

The last step was to release the phenolic and the sulfamate derivatives from the solid support by either a nucleophilic or an acid treatment, respectively. The nucleophilic cleavage was done first because the phenolic derivatives are not totally released from the solid support under these conditions. Furthermore, E_2_ derivatives are preferred since building blocks were chosen to target 17β-HSD1. Thus, resins **28** were treated with a solution of 30% diethylamine (DEA) in THF at room temperature for 24 to 66 h to release E_2_ derivatives **59–88** (library B) and **89–151** (library C). After the nucleophilic treatment, the resin was washed with acetone or THF and MeOH for libraries B and C, respectively. The filtrate was evaporated. The crude product was dissolved in EtOAc or THF and evaporated again. This procedure was done twice in order to provide DEA-free E_2_ derivatives. The acid cleavage was performed on resins **28** that were previously treated by a nucleophilic cleavage for 24 h to generate library B. In fact, there were still steroids loaded on resins **28** after the nucleophilic cleavage. Thus, these resins **28** were treated with a solution of 30% hexafluoroisopropanol (HFIP) in DCM for 6 h at room temperature. Evaporation of the solvent afforded sulfamoylated E_2_ derivatives **29–58** (library A). The acid cleavage was not carried on with 5% TFA in DCM to avoid the formation of C17 trifluoroacetate derivative as previously reported in the preparation of a 17α-substituted E_2_ sulfamate library [31].

### 2.4. Characterization of libraries A, B and C (Table 1, Table 2 and Table 3)

Sulfamoylated E_2_ derivatives of library A and E_2_ derivatives of libraries B and C were submitted to a random sampling after we confirmed the presence of a major compound by TLC analysis. Six members of library A, six members of library B and 14 members of library C were then characterized by ^1^H-NMR and LRMS. In addition, the purity of the sampling of libraries A and B was determined by HPLC analysis. Expected masses matched for all these compounds. Furthermore, ^1^H-NMR analysis confirmed the structure of the library members. The HPLC-purity of final compounds ranged from 89 to 91% and 85 to 95% for libraries A and B, respectively. The average HPLC-purity was 90% for both libraries. Results were very satisfactory because no purification step was performed after the final cleavage. The excess of reagent (HFIP and DEA) and solvent were simply removed by evaporation. The average overall yields for the solid-phase sequence of reactions (6 or 7 steps for libraries A and B, and 9 steps for library C) were 46%, 27% and 31%, respectively. The average overall yield was calculated after the loading step of precursor **6a** or **6b** on trityl chloride resin. The mass of phenols released from solid-support by nucleophilic cleavage to provide library B was not removed in the calculation of the overall yield of library A. The purity of library C members was estimated to be around 70–75% by TLC and ^1^H-NMR analysis. The lower purity of library C members could be explained by the final deprotection step on solid-phase. In fact, resins **27** were treated with a solution of MeONa in MeOH and THF for approximately 100 h instead of 48 h in order to complete the reaction. However, this long treatment resulted in degradation of part of the product. Nevertheless, the purity of library C members was found sufficiently satisfactory for screening assay on 17β-HSD1.

### 2.5. Chemical synthesis of compound ***156*** used in the preparation of library D (Scheme 4)

The capping groups of members from library D have a carboxylic acid as functional group to interact with the cofactor (adenosine) binding site of 17β-HSD1. In fact, among the simplified bisubstrate compounds prepared to inhibit this enzyme, carboxylic acid derivatives were found to be by far the best inhibitors of that series [36]. The introduction of the capping building block on solid-phase was done by a reaction between an amine and a carboxylic acid. Indeed, the capping synthon **156** is a dicarboxylic acid and one of the COOH group has to be appropriately protected. The *tert*-butyl ester was chosen as protective group because it could be easily removed in acid conditions after the nucleophilic cleavage. Thus, starting from commercially available 2-(3-bromophenyl)-acetic acid (**152**), the carboxylic acid was protected with di-*tert*-butyl dicarbonate and dimethylaminopyridine (DMAP) in *tert*-butanol to afford *tert*-butyl ester **153** in an excellent 94% yield [37]. A vinyl was next added on aryl bromide **153** by a Stille reaction using mild conditions (tributylvinyl tin, Pd_2_(dba)_3_, P(*t*-Bu)_3_ in toluene) to afford **154** in 88% yield [38]. Hydroboration of vinyl **154** using standard condition provided alcohol **155**. This alcohol was oxidized under Jones’ conditions in order to give carboxylic acid **156**. However, the reaction conditions were too drastic and afforded the dicarboxylic acid product instead of **156**. A 2-step procedure was then necessary to avoid hydrolysis of the *tert*-butyl ester. Thus, alcohol **155** was first oxidized with Dess-Martin periodinane to provide the aldehyde, which was subsequently oxidized in carboxylic acid using mild conditions (NaClO_2_, NaH_2_PO_4_ and 2-methyl-2-butene in *t*-BuOH). Thus, carboxylic acid **156** was synthesized in five steps and 26% overall yield.

### 2.6. Solid-phase synthesis of library D (Scheme 5)

The preparation of library D required a series of Fmoc-protected amino acids with a functionalised side chain as building blocks instead of a hydrophobic one. Therefore, these amino acids needed an appropriate protective group. A *tert*-butyl ester and a trityl group were chosen as protective groups of the amino acid side chain since they could be easily removed in acid conditions as for the *tert*-butyl ester found on the capping building block. Furthermore, the molecular diversity was limited to the introduction of two amino acids. The same carboxylic acid **156** was introduced on each member of library D. Thus, focus was put on the interactions with the left part (nicotinamide and the spacer) of the cofactor-binding site. Library D was prepared as previously described for libraries B and C. Briefly, sulfamate precursor **6b** was loaded on trityl chloride resin. The loading yield of **6b** on the resin was 40%. Since three levels of diversity were introduced on the steroid scaffold, 1 equivalent of precursor **6b** was used for 2 equivalents of trityl chloride resin as for library C. The two levels of molecular diversity were then introduced by coupling Fmoc-protected amino acids on resin **158**. Fmoc-*L*-Phe-OH, Fmoc-*L*-4-Pal-OH or Fmoc-*L*-Ala-OH, Fmoc-*L*-Asp(O*t*Bu)-OH, Fmoc-*L*-Ser(Trt)-OH and Fmoc-*L*-Tyr(2-Cl-Trt)-OH were selected as building blocks for both levels. The carboxylic acid **156** was introduced as a capping group on resins **162** in DMF and DIPEA, using PyBrOP and HOBt as coupling reagent. Removal of the C17-THP and the trityl protective group in one step on resin **163** was tried but without success. Thus, the trityl and the 2-chloro-trityl were removed by two 10-minute treatments with a solution of TFA/TIS(triisopropylsilane)/ DCM (2:1:97). The C17-THP protective group was next taken off with a solution of *p*-TSA in DCM and *t*-BuOH. Phenols were then released from the solid support under nucleophilic conditions (30% DEA/THF) for 65 h. Next, *tert*-butyl ester of compounds **165** was hydrolysed under acid conditions. Reaction of esters **165** with a solution of HCl (4M) in dioxane for 3 h was found to give the best conditions of hydrolysis. Evaporation of the reaction mixture provided carboxylic acids **166–190**. However, these carboxylic acids were not pure enough for screening assay on 17β-HSD1. For this purpose, crude carboxylic acids (dried for at least 16 h under a vacuum) were dissolved in MeOH and preadsorbed on C-18 silica gel. They were subsequently purified by flash chromatography on C18-silica gel (reverse phase) with a mixture of H_2_O and MeOH as eluent.

### 2.7. Characterization of library D (Table 4) and discussion

All products were characterized by LRMS and ^1^H-NMR and the results are presented in Table 4. The molecular ion peak was found in LRMS for all expected compounds (except for compounds **174** and **181**) and ^1^H-NMR analysis reveals the presence of the steroid nucleus and the molecular diversity. However, only six compounds were obtained as carboxylic acid derivatives. In fact, the presence of numerous peaks in the region of 3.4 to 3.7 ppm in the ^1^H-NMR revealed that methyl ester contaminated the carboxylic acid products in different proportions for several compounds. Furthermore, a peak corresponding to [M+H+14]^+^ or [M-H+14]^-^ was found in almost all LRMS spectra. We found that the methyl ester was produced during the preabsorption process on silica gel using MeOH as solvant. Even if the carboxylic acids were dried under a vacuum for 16 h after the hydrolysis step, traces of HCl trapped in the product seem to be enough to promote the formation of the methyl ester. Unfortunately, the carboxylic acid function was very important for the interactions with the cofactor (adenosine) - binding site. For this reason, carboxylic acids **166–190** were not tested as inhibitors of 17β-HSD1.

The average overall yield for the preparation of library D (9 solid-phase steps and 1 solution-phase step) was 16% after purification. The lower overall yield was generally obtained with serine derivatives, which seem to be less tolerant under acid conditions during the final deprotection step. In fact, an important proportion of a less polar compound was observed but could not be identified.

The main problem during the preparation of library D occurred with the hydrolysis of the *tert*-butyl ester. After the nucleophilic cleavage step, the products were obtained with a good purity estimated to be higher than 80% by TLC analysis of all members and ^1^H-NMR analysis of two compounds (**165**: R_1_ = a and R_2_ = e; **165**: R_1_ = c and R_2_ = d). The choice of protective groups for this library was based on the fact that trityl and *tert*-butyl ester could be easily removed with TFA/H_2_O/TIS (95:2.5:2.5) and the product obtained should precipitate in diethyl ether as described in the note from the NovaBiochem catalogue. Even if this procedure works well for peptide synthesis, the acid conditions were too drastic for our steroid derivatives, resulting in decomposition of the product. Knowing this fact, a new chemical strategy could be used for the preparation of library D. The main difference would be the structure of the building block for the capping. Instead of using a *tert*-butyl ester to protect the carboxylic acid, a methyl ester could be used. It is obvious that aspartic acid used as a building block should also be protected as a methyl ester. Thus, the solid-phase synthesis would be very similar to the preparation of library D and the methyl ester would be hydrolysed at the last solid-phase step, using a solution of NaOH in THF. This procedure was previously described for the hydrolysis of an amino triflate loaded on solid support by the sulfamate linker.^31^ Using this method, or alternatively a benzyl ester as protective group, library D members should be obtained in good purity without any purification.

### 2.8. Inhibition of steroid sulfatase by sulfamoylated E_2_ derivatives from library A

The inhibitory potential of the members of library A on the steroid sulfatase was tested and the results were presented in our early report [30]. A sulfamate on position C3 of the steroid is an essential functional group to obtain a potent steroid sulfatase inhibition [39]. Among the 30 sulfamoylated E_2_ derivatives of this library, compound **34** gave a better inhibition than estrone sulfamate (EMATE), a well known and potent inhibitor of steroid sulfatase [40]. The polar building blocks were however chosen to target 17β-HSD1 instead of steroid sulfatase, the inhibitory result could thus be better when using hydrophobic groups as elements of molecular diversity. In fact, potent inhibitors of steroid sulfatase were obtained when hydrophobic groups were added on the C16- or C17-position of E_2_ sulfamate [30,31,32].

### 2.9. Inhibition of 17β-HSD1 by E_2_ derivatives from libraries B and C

Inhibition of the transformation of [^14^C]-E_1_ into [^14^C]-E_2_ by members from libraries B and C was tested on homogenated human embryonic kidney (HEK)-293 cells transfected with a vector encoding for 17β-HSD1 according to a previously reported procedure [15,41]. Briefly, the enzymatic assay was conducted for 2 h at 37 °C in a sodium phosphate buffer (pH = 7.4) with NADH as cofactor.

**Figure 4:**
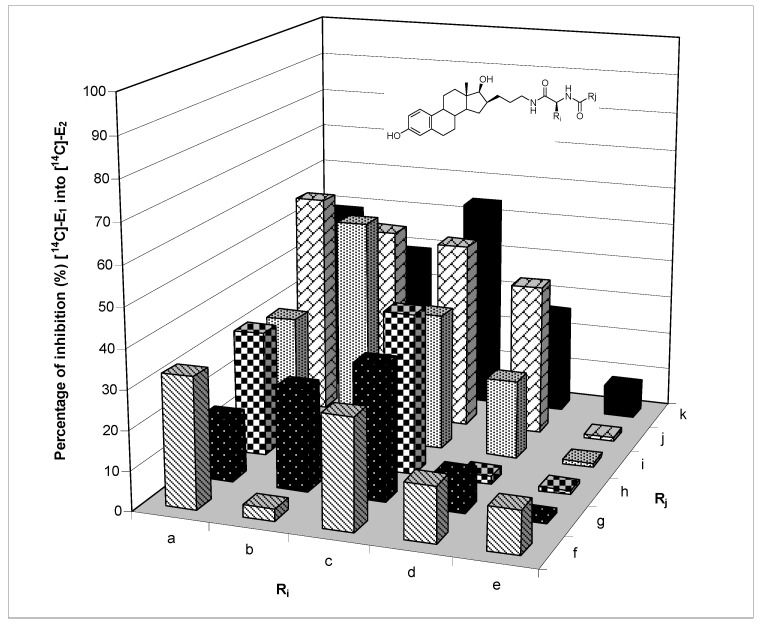
Inhibition of the transformation of [^14^C]-E_1_ (0.1 µM) into [^14^C]-E_2_ in homogenated HEK-293 cells overexpressing 17β-HSD1 by E_2_ derivatives of library B (compounds **59–88**) at 1 µM. The letters a-k represent the residue (R_i_ and R_j_) on side chain (see Scheme 3). See experimental section for more details on this enzymatic assay.

The screening assay on 17β-HSD1 for members of library B (Table 2) is presented in Figure 4. There are two levels of molecular diversity in this library. Hydrophobic amino acids were mainly chosen for the first level to avoid the protective group on amino acid side chains. Pyridine, phenyl, aniline, pyrazine, 3-aminopyrazine and indole nucleus were selected for the second level of molecular diversity. These capping building blocks were chosen to mimic the adenosine moiety found in EM-1745 (**1**), one of the most potent inhibitors of 17β-HSD1 [20]. For library B, the best inhibitions were obtained when phenylalanine was used as a building block for the first level with 16 to 57% at 1 µM for compounds **59–64**. In general, lower inhibitions were obtained with isoleucine and proline (compounds **65–76**) and almost no inhibition was observed when alanine and glycine were used as building blocks for the first level (compounds **77–88**). For the second level of molecular diversity, better inhibitions were usually obtained with pyrazine, 2-aminopyrazine and indole moieties. However, these compounds weren’t more potent inhibitors than unlabeled-E_1_ (59% at 1 µM) and EM-251 (52% at 1 µM), a known inhibitor of 17β-HSD1 used as reference compound [42]. They were also no more potent than EM-1745 (**1**, 97% at 1 µM).

**Figure 5:**
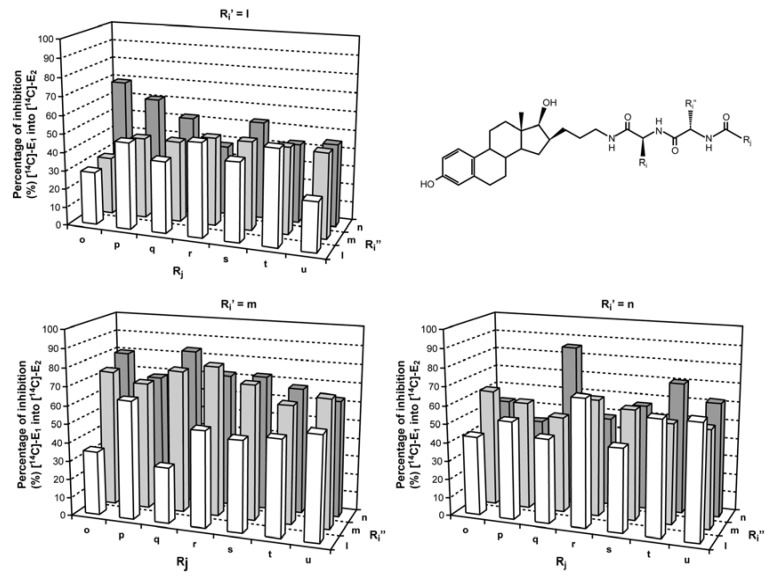
Inhibition of the transformation of [^14^C]-E_1_ (0.1 µM) into [^14^C]-E_2_ in homogenated HEK-293 cells overexpressing 17β-HSD1 by E_2_ derivatives of library C (compounds **89–151**) at 1 µM. The letters l-u represent the residue (R_i_′, R_i_″ and R_j_) on side chain (see Scheme 3). See the experimental section for more details on this enzymatic assay.

Therefore, in order to improve the inhibitory potency of library B members, another library was prepared. The members of library C have longer spaces between the C16β E_2_ and the polar capping building block. This way, the capping was able to interact with the cofactor (adenosine) binding site. Fmoc-*L*-Phe-OH and Fmoc-*L*-4-NO_2_-Phe-OH were chosen as building blocks for the first and second level of molecular diversity because members of library B with a phenylalanine as building block gave the best inhibitors. Glycine was also chosen but should give less potent inhibitors. Aniline derivatives (compounds **12–18**) were selected as building blocks for the third level of molecular diversity. In fact, the aniline should form interactions with Asp65 such as the amine of the adenosine moiety of EM-1745 does in the cofactor-binding site [21]. Members of library C were tested to determine their potential to inhibit the enzyme activity at 1 µM and the results are presented in Figure 5. Three points can be highlighted from these results. First, better inhibitions were generally obtained with three levels of molecular diversity (library C) than with two levels (library B). In fact, the average percentage of inhibition for library B is 26% while for library C it is 56%. Second, the best inhibitors were obtained when phenylalanine was used as a building block for the first level of molecular diversity (R_i_’ = m, compounds **110–130**) with more than half of the compounds giving better inhibition than EM-251 (59% at 1 µM) and eight compounds giving a better inhibition than unlabeled-E_1_ (73% at 1 µM). Good inhibitions were also obtained with 4-nitro-phenylalanine used as building block for the first or the second level of molecular diversity. However, as expected, lower inhibition was obtained with glycine. Third, similar inhibitions were found regardless of which aniline building blocks were selected for the third level. Globally, 26 of the 63 members of library C gave better inhibition than EM-251, and eight compounds (**117**, **119**, **120**, **121**, **124**, **126**, **128** and **147**) gave a better inhibition than E_1_.

## 3. Conclusions

We have successfully used the sulfamate linker to prepare one library of sulfamoylated E_2_ derivatives and two libraries of E_2_ derivatives by parallel solid-phase synthesis. Precursor **6a** was loaded on trityl chloride resin via the sulfamate linker and two or three levels of molecular diversity were introduced using Fmoc-protected amino acids or carboxylic acids as building blocks. Acid (HFIP/THF) or nucleophilic (DEA/THF) cleavage respectively released the sulfamoylated E_2_ derivatives (library A) or the E_2_ derivatives (library B) from the resin in excellent HPLC purity (90% for libraries A and B). However, E_2_ derivatives from library C were obtained with a lower purity (around 70–75%) estimated by TLC and ^1^H-NMR analysis. Members of libraries B and C were also screened on homogenated HEK-293 cells overexpressing 17β-HSD1. Compounds of library C, with three levels of molecular diversity, gave better inhibition than E_2_ derivatives of library B. Furthermore, 26 compounds gave a better inhibition than EM-251, a known inhibitor of 17β-HSD1 and 8 of these compounds gave a better inhibition than E_1_, used as an inhibitor. In both libraries, better inhibitors where those bearing a phenylalanine as building block. The eight top inhibitors of library C should be evaluated as pure compounds on 17β-HSD1 in order to confirm the potential of these inhibitors. However, none of the members of both libraries gave a better inhibition than EM-1745, one of the most potent inhibitors of this enzyme. In order to obtain more potent inhibitors, a third library of E_2_ derivatives (library D) was also prepared as described above. However, Fmoc-protected amino acids with functionalised side-chain and a protected carboxylic acid were used as building blocks. Unfortunately, the final hydrolysis of *tert*-butyl ester did not work as well as expected and methyl ester contaminated the final carboxylic acid products. The use of a methyl or a benzyl ester as protective group instead of a *tert*-butyl ester is suggested as a mean to avoid the final purification step. It is also expected that the E_2_ derivatives reported in library D will more closely mimic the EM-1745/17β-HSD1 interactions than those reported in libraries A-C, thus resulting in better 17β-HSD1 inhibition.

## 4. Experimental

### 4.1. General

Trityl chloride resin, Fmoc-OSu, coupling reagents and Fmoc amino acids were supplied by EMD Biosciences (Novabiochem, La Jolla, CA, USA) or Advanced ChemTech (Louisville, KY, USA). Other reagents were obtained from Sigma-Aldrich Canada Co. (Oakville, ON, Canada). Usual solvents were obtained from Fisher Scientific (Montréal, QC, Canada) and VWR (Ville Mont-Royal, QC, Canada) and were used as received. Anhydrous solvents were purchased from Aldrich and VWR in SureSeal bottles, which were kept under positive argon pressure. Tetrahydrofuran (THF) was distilled from sodium/benzophenone under argon. Solution-phase reactions were performed under positive argon pressure in oven-dried glassware with magnetic stirring bars. Fritted peptide synthesis vessels (peptide flasks, 25 or 50 mL) equipped for vacuum filtration (ChemGlass Inc, Vineland, NJ) were used for the solid-phase synthesis on a Burrell wrist-action shaker model 75 (Burrell, Pittsburgh, PA). The libraries of steroid derivatives were realized with ACT LabTech manual synthesizer (Advanced ChemTech, Louisville, KY, USA) using either a 40 or a 96 solid-phase reaction block. Thin-layer chromatography (TLC) was performed on 0.25-mm silica gel 60 F_254_ plates (Whatman, Maidstone, England), and compounds were visualized by exposure to UV light (254 nm), with a solution of ammonium molybdate/sulphuric acid/water (with heating) or with a solution of *p*-anisaldehyde (with heating). Flash chromatography was performed on Silicycle 60 (Québec, QC, Canada) 230–400 mesh silica gel. The purity of a random sampling of libraries A and B members was determined by HPLC (Waters Associates, Milford, MA, USA) using a NovaPak C_18_ reversed-phase column (150 × 3.9 mm id) and an ultra-violet detector (205 or 210 nm). The eluent for HPLC was a mixture of 50% of MeOH/H_2_O (90:10) and 50% of H_2_O, both containing 20 mM of NH_4_OAc. IR spectra were obtained neat, from KBr pellet or from a thin film of the solubilized compound on NaCl pellet (usually in CH_2_Cl_2_). They were recorded on a Perkin-Elmer series 1600 FT-IR spectrometer (Norwalk, CT, USA); only significant bands are reported (in cm^−1^). ^1^H- and ^13^C-NMR spectra were recorded with a Bruker AC/F 300 spectrometer (Billerica, MA, USA) at 300 and 75 MHz, respectively, and a Bruker AVANCE 400 spectrometer at 400 (^1^H) and 100 (^13^C) MHz. The chemical shifts (δ) are expressed in ppm and referenced to chloroform (7.26 and 77.0 ppm), acetone (2.07 and 206.0 ppm), methyl sulfoxide (2.51 and 39.5 ppm) or methanol (3.33 and 49.0 ppm) for ^1^H and ^13^C respectively. Duplication of NMR signals was generally recorded for THP derivatives (presence of 2 stereoisomers). These 2 stereoisomers increased the complexity of ^13^C-NMR spectra, and additional peaks are written in parentheses. Assignment of NMR signals was made easier using literature data [43]. Low-resolution mass spectra (LRMS) were recorded with an LCQ Finnigan apparatus (San Jose, CA, USA) equipped with an atmospheric pressure chemical ionisation (APCI) source on positive or negative mode.

### 4.2. Chemical synthesis

#### 4.2.1. 3-Sulfamoyloxy-16β-[3-(9H-fluoren-9-ylmethoxycarbonylamino)-propyl]-17β-acetoxy-estra-1,3,5(10)-triene (**6a**)

The starting material 3-(3-*tert*-butyldimethylsilyloxy-17β-acetoxy-estra-1,3,5(10)-trien-16β-yl)-azido-propane (**4a**) was synthesized as previously reported [33]. A suspension of **4a** (680 mg, 1.32 mmol) and 5% Pd/C (136 mg) in EtOAc (15 mL) and MeOH (160 mL) was stirred under a hydrogen atmosphere at room temperature. After 2 h, the resulting suspension was filtered through Celite, washed with MeOH and evaporated to dryness to afford the amine (620 mg, 96%) in the form of a viscous colourless oil in good purities without purification. To a solution of the crude amine (620 mg) in anhydrous THF (40 mL) under an argon atmosphere at 0 °C was added a solution of TBAF (1M in THF, 1.53 mL, 1.53 mmol). After 30 min, the reaction was quenched by addition of a saturated aqueous solution of NaHCO_3_. The crude product was extracted with EtOAc and the organic phase was washed with brine, dried over MgSO_4_ and evaporated to dryness to afford the phenol (585 mg) in the form of a yellowish solid. The crude phenol (500 mg) was dissolved in THF (120 mL) and H_2_O (40 mL). At 0 °C, NaHCO_3_ (1M in H_2_O, 1.84 mL, 1.84 mmol) and Fmoc-OSu (570 mg, 1.69 mmol) were added and the reaction was stirred for 1 h at 0 °C. Then, the reaction mixture was quenched by addition of H_2_O and the crude product was extracted with EtOAc and DCM. Each organic phase was washed with brine, dried over MgSO_4_, and evaporated to dryness to provide **5a** (1.05 g) in the form of a white foam. To a solution of crude phenol **5a** (1.05 g) in dry DCM (120 mL) under an argon atmosphere were added 2,6-di-*t*-butyl-4-methylpyridine (933 mg, 4.55 mmol) and NH_2_SO_2_Cl [34] (421 mg, 3.64 mmol). The reaction was stirred for 24 h at room temperature and quenched by the addition of ice water. The crude product was extracted with DCM and the organic phase was washed with brine, dried over MgSO_4_, and evaporated to dryness. Purification by flash chromatography (hexanes/EtOAc, 6:4) gained precursor **6a** (604 mg, 82% yield for 4 steps) in the form of a white foam. IR (film) 3393 (NH and NH_2_), 1716 (C=O, ester and carbamate), 1374 and 1188 (S=O, sulfamate); ^1^H-NMR (300 MHz, CDCl_3_) δ 0.80 (s, 18-CH_3_), 1.00 to 2.35 (m, CH and CH_2_ of steroid skeleton and alkyl chain), 2.07 (s, CH_3_CO), 2.84 (m, 6-CH_2_), 3.18 (m, CH_2_NH), 4.20 (t, *J* = 6.8 Hz, CH_2_CH of Fmoc), 4.38 (d, *J* = 6.9 Hz, CH_2_CH of Fmoc), 4.73 (d, *J* = 10.0 Hz, 17α-CH), 4.76 (m, NHFmoc), 5.01 (s_br_, NH_2_), 7.01 (s, 4-CH), 7.05 (d, *J_1_* = 8.6 Hz, *J_2_* = 2.3 Hz, 2-CH), 7.28 (m, 1-CH and 2 × CH of Fmoc), 7.38 (t, *J* = 7.4 Hz, 2 × CH of Fmoc), 7.57 (d, *J* = 7.4 Hz, 2 × CH of Fmoc), 7.75 (d, *J* = 7.4 Hz, 2 × CH of Fmoc); ^13^C-NMR (75 MHz, CDCl_3_) δ 13.3, 21.0, 25.9 (2×), 27.0, 28.8, 29.5, 32.1, 37.5, 37.6, 38.2, 41.1, 43.4, 43.9, 47.3, 48.7, 66.6, 83.2, 118.9, 120.0 (2×), 121.9, 125.0 (2×), 126.8, 127.0 (2×), 127.7 (2×), 138.8, 139.5, 141.3 (2×), 144.0 (2×), 147.9 156.4, 171.2; LRMS calculated for C_38_H_43_N_2_O_7_S [M-H]^-^ 671.3, found 671.1 m/z.

#### 4.2.2. 3-Sulfamoyloxy-16β-[3-(9H-fluoren-9-ylmethoxycarbonylamino)-propyl]-17β-(tetrahydro-2H-pyran-2-yl-oxy)-estra-1,3,5(10)-triene (**6b**)

3-(3-Hydroxy-17β-(tetrahydro-*2H*-pyran-2-yl-oxy)-estra-1,3,5(10)-trien-16β-yl)-azidopropane (**4b**) was synthesized as previously reported [33]. A suspension of **4b** (2.35 g, 5.35 mmol) and 5% Pd/C (470 mg) in MeOH (535 mL) was stirred under hydrogen atmosphere at room temperature. After 3 h, the resulting suspension was filtered through celite, washed with MeOH and evaporated to dryness to afford the amine (2.00 g) in good purities without purification. The crude amine (2.00 g) was dissolved in THF (360 mL) and H_2_O (120 mL). At 0 °C, NaHCO_3_ (1M in H_2_O, 6.3 mL, 6.3 mmol) and Fmoc-OSu (1.96 g, 5.81 mmol) were added and the reaction was stirred for 1 h at 0 °C. The reaction mixture was then quenched by addition of H_2_O and the crude product was extracted with EtOAc and DCM. Each organic phase was washed with brine, dried over MgSO_4_, and evaporated to dryness to provide **5b** (3.62 g) in the form of a white foam. To a solution of crude phenol **5b** (3.62 g) in dry DCM (280 mL) under an argon atmosphere were added di-*tert*-butyl-4-methylpyridine (2.92 g, 14.2 mmol) and NH_2_SO_2_Cl [34] (1.31 g, 11.4 mmol). The reaction was stirred for 16 h at room temperature and quenched by addition of icewater. The crude product was extracted with DCM and the organic phase was washed with brine, dried over MgSO_4_ and evaporated to dryness. Purification by flash chromatography (hexanes/EtOAc, 8:2 to 6:4) provided precursor **6b** (2.00 g, 52% yield for 3 steps) as a white foam. The 17β-unprotected precursor (1.27 g) was also obtained. This product was reprotected with a THP using this procedure. The 17β-hydroxy precursor (1.27 g, 2.07 mmol) was dissolved in a minimum of dry THF (~2 mL) and DCM (40 mL) under an argon atmosphere at 0 °C. Then, 3,4-dihydro-2*H*-pyran (187 µL, 2.07 mmol) and *p*-TSA (39 mg, 0.21 mmol) were added and the reaction was stirred for 10 min. The reaction was quenched by addition of saturated aqueous solution of NaHCO_3_ and the crude precursor was extracted with DCM. The organic phase was washed with brine, dried over MgSO_4_ and evaporated under reduced pressure. Purification by flash chromatography (hexanes/EtOAc, 7:3 to 6:4) afforded precursor **6b** (1.09 g, 74% yield). IR (film) 3392 (NH and NH_2_), 1698 (C=O, carbamate), 1386 and 1187 (S=O, sulfamate); ^1^H-NMR (400 MHz, acetone-d_6_) δ 0.83 and 0.87 (2s, 18-CH_3_), 1.00 to 2.40 (m, CH and CH_2_ of steroid skeleton and alkyl chain), 2.87 (m, 6-CH_2_), 3.19 (m, CH_2_NH), 3.47 and 3.92 (2m, OCH_2_ of THP), 3.76 and 3.80 (2d, *J* = 10.0 Hz, 17α-CH), 4.23 (t, *J* = 7.1 Hz, CH_2_CH of Fmoc), 4.33 (d, *J* = 7.2 Hz, CH_2_CH of Fmoc), 4.64 and 4.71 (2m, CH of THP), 6.52 (NH), 7.08 (m, 2-CH, 4-CH and NH_2_), 7.34 (m, 1-CH and 2 × CH of Fmoc), 7.42 (t, *J* = 7.4 Hz, 2 × CH of Fmoc), 7.71 (d, *J* = 7.5 Hz, 2 × CH of Fmoc), 7.88 (d, *J* = 7.5 Hz, 2 × CH of Fmoc); ^13^C-NMR (100 MHz, acetone-d_6_) δ 13.5 (14.3), 20.0 (20.5), 26.2 (26.3), 26.77, 26.84, 27.7, 29.1, 29.3 to 30.2 (1C under solvent peaks), 31.3 (31.6), 32.78 (32.82), 38.6, 38.70 (38.74), 39.0 (40.2), 41.5 (41.6), 44.2 (44.7), 44.6 (44.8), 48.0, 49.3 (49.5), 62.0 (63.0), 66.5, 86.1 (86.6), 98.2 (99.7), 120.0, 120.6 (2×), 122.8, 125.9 (2×), 127.2, 127.7 (2×), 128.5 (2×), 139.0, 139.6, 141.9 (2×), 145.0 (2×), 149.1, 156.9; LRMS calculated for C_41_H_49_N_2_O_7_S [M-H]^-^ 713.3, found 713.3 m/z.

#### 4.2.3. General procedure to synthesize Fmoc-NH protected carboxylic acid building blocks **14–18**

Amines **9** and **10** were prepared as previously reported [35]. Amines **7–11** (0.90–1.15 g, 5.45–6.93 mmol) were dissolved in THF (50 mL) and H_2_O (10 mL). Then, NaHCO_3_ (1M in H_2_O, 13.6–17.3 mL, 13.6–17.3 mmol) and Fmoc-OSu (2.76–3.51 g, 8.17–10.4 mmol) were added. The reaction was stirred for 16 h at room temperature. The reaction mixture was then preabsorbed on silica gel and purified by flash chromatography (DCM/MeOH, 95:5 to 85:15) to afford **14–18** (1.35–2.00 g, 51–93% yield).

*2-[4′-(9H-Fluoren-9-ylmethoxycarbonylamino)-phenyl]-acetic acid* (**14**). White solid (1.72 g, 70% yield); IR (KBr) 3700–2200 (OH, COOH), 3336 (NH), 1708 (C=O, acid and carbamate); ^1^H-NMR (400 MHz, DMSO-d_6_) δ 3.47 (s, CH_2_COOH), 4.31 (t, *J* = 6.5 Hz, CH_2_CH of Fmoc), 4.49 (d, *J* = 6.2 Hz, CH_2_CH of Fmoc), 7.16 (d, *J* = 7.8 Hz, 2′-CH and 6′-CH), 7.39 (m, 3′-CH, 5′-CH and 4 × CH of Fmoc), 7.76 (d, *J* = 7.3 Hz, 2 × CH of Fmoc), 7.91 (d, *J* = 7.6 Hz, 2 × CH of Fmoc), 9.69 (s_br_, NH); ^13^C-NMR (75 MHz, DMSO-d_6_) δ 40.3, 46.7, 65.5, 118.2 (2×), 120.2 (2×), 125.2 (2×), 127.1 (2×), 127.7 (2×), 129.5, 129.7 (2×), 137.5, 140.8 (2×), 143.8 (2×), 153.5, 173.2; LRMS calculated for C_23_H_18_NO_4_ [M-H]^-^ 372.1, found 372.2 m/z.

*2-[3′-(9H-Fluoren-9-ylmethoxycarbonylamino)-phenyl]-acetic acid* (**15**). White solid (2.00 g, 81% yield); IR (KBr) 3600–2200 (OH, COOH), 3287 (NH), 1702 (C=O, acid and carbamate); ^1^H-NMR (400 MHz, DMSO-d_6_) δ 3.51 (s, CH_2_COOH), 4.32 (t, *J* = 6.7 Hz, CH_2_CH of Fmoc), 4.47 (d, *J* = 6.6 Hz, CH_2_CH of Fmoc), 6.90 (d, *J* = 7.5 Hz, 6′-CH), 7.21 (t, *J* = 7.7 Hz, 5′-CH), 7.40 (m, 2′-CH, 4′-CH and 4 × CH of Fmoc), 7.77 (d, *J* = 7.4 Hz, 2 × CH of Fmoc), 7.92 (d, *J* = 7.4 Hz, 2 × CH of Fmoc), 9.74 (s_br_, NH), 12.37 (s_br_,COOH); ^13^C-NMR (75 MHz, DMSO-d_6_) δ 41.0, 46.6, 65.6, 116.7, 119.2, 120.2 (2×), 123.6, 125.2 (2×), 127.2 (2×), 127.7 (2×), 128.6, 135.6, 139.0, 140.8 (2×), 143.8 (2×), 153.4, 172.6; LRMS calculated for C_23_H_20_NO_4_ [M+H]^+^ 374.1, found 374.1 m/z.

*3-[4′-(9H-Fluoren-9-ylmethoxycarbonylamino)-phenyl]-propionic acid* (**16**). White solid (1.35 g, 51% yield); IR (KBr) 3500–2000 (OH, COOH), 3331 (NH), 1702 (C=O, acid and carbamate); ^1^H-NMR (400 MHz, DMSO-d_6_) δ 2.50 (t, *J* = 7.5 Hz, CH_2_COOH), 2.76 (t, *J* = 7.5 Hz, CH_2_CH_2_COOH), 4.31 (t, *J* = 6.5 Hz, CH_2_CH of Fmoc), 4.48 (d, *J* = 6.3 Hz, CH_2_CH of Fmoc), 7.13 (d, *J* = 7.9 Hz, 2′-CH and 6′-CH), 7.39 (m, 3′-CH, 5′-CH and 4 × CH of Fmoc), 7.76 (d, *J* = 7.4 Hz, 2 × CH of Fmoc), 7.91 (d, *J* = 7.4 Hz, 2 × CH of Fmoc), 9.65 (s_br_, NH), 12.13 (s_br_, COOH); ^13^C-NMR (75 MHz, DMSO-d_6_) δ 29.7, 35.4, 46.6, 65.5, 118.4 (2×), 120.2 (2×), 125.1 (2×), 127.1 (2×), 127.7 (2×), 128.5 (2×), 134.9, 137.0, 140.8 (2×), 143.8 (2×), 153.4, 173.8; LRMS calculated for C_24_H_22_NO_4_ [M+H]^+^ 388.1, found 388.0 m/z.

*3-[3′-(9H-Fluoren-9-ylmethoxycarbonylamino)-phenyl]-propionic acid* (**17**). White solid (1.96 g, 93% yield); IR (KBr) 3500–2000 (OH, COOH), 3307 (NH), 1696 (C=O, acid and carbamate); ^1^H-NMR (400 MHz, DMSO-d_6_) δ 2.52 (t, *J* = 7.5 Hz, CH_2_COOH), 2.79 (t, *J* = 7.4 Hz, CH_2_CH_2_COOH), 4.32 (t, *J* = 6.6 Hz, CH_2_CH of Fmoc), 4.48 (d, *J* = 6.3 Hz, CH_2_CH of Fmoc), 6.87 (d, *J* = 7.5 Hz, 6′-CH), 7.18 (t, *J* = 7.7 Hz, 5′-CH), 7.40 (m, 2′-CH, 4′-CH and 4 × CH of Fmoc), 7.77 (d, *J* = 7.4 Hz, 2 × CH of Fmoc), 7.91 (d, *J* = 7.5 Hz, 2 × CH of Fmoc), 9.70 (s_br_, NH), 12.18 (s_br_, COOH); ^13^C-NMR (75 MHz, DMSO-d_6_) δ 30.5, 35.2, 46.7, 65.6, 116.2, 118.2, 120.2 (2×), 122.4, 125.2 (2×), 127.2 (2×), 127.7 (2×), 128.7, 139.1, 140.8 (2×), 141.5, 143.8 (2×), 153.4, 173.7; LRMS calculated for C_24_H_22_NO_4_ [M+H]^+^ 388.1, found 388.0 m/z.

*4-[4′-(9H-Fluoren-9-ylmethoxycarbonylamino)-phenyl]-butanoic acid* (**18**). White solid (1.59 g, 68% yield); IR (KBr) 3500–2000 (OH, COOH), 3345 (NH), 1708 (C=O, acid and carbamate); ^1^H-NMR (400 MHz, DMSO-d_6_) δ 1.77 (quintuplet, *J* = 7.5 Hz, CH_2_CH_2_COOH), 2.20 (t, *J* = 7.4 Hz, CH_2_COOH), 2.52 (m, CH_2_CH_2_CH_2_COOH), 4.31 (t, *J* = 6.5 Hz, CH_2_CH of Fmoc), 4.48 (d, *J* = 6.2 Hz, CH_2_CH of Fmoc), 7.09 (d, *J* = 7.9 Hz, 2′-CH and 6′-CH), 7.39 (m, 3′-CH, 5′-CH and 4 × CH of Fmoc), 7.76 (d, *J* = 7.4 Hz, 2 × CH of Fmoc), 7.91 (d, *J* = 7.5 Hz, 2 × CH of Fmoc), 9.65 (s_br_, NH), 12.07 (s_br_, COOH); ^13^C-NMR (75 MHz, DMSO-d_6_) δ 26.4, 33.0, 33.7, 46.7, 65.5, 118.4 (2×), 120.2 (2×), 125.2 (2×), 127.1 (2×), 127.7 (2×), 128.6 (2×), 135.6, 136.9, 140.8 (2×), 143.8 (2×), 153.5, 174.3; LRMS calculated for C_25_H_24_NO_4_ [M+H]^+^ 402.2, found 402.0 m/z.

#### 4.2.4. Synthesis of resin **20** for preparation of libraries A and B

The loading reaction was run in two 25 mL peptide flasks using the same amount of resin. In each peptide flask, a solution of precursor **6a** (1.07 g, 1.62 mmol) in dry DCM (10 mL) was added to trityl chloride resin (1.09 g, 1.24 mmol/g theoretical loading) under an argon atmosphere. After 5 min, diisopropylethylamine (DIPEA, 2.16 mL) was added and the mixture was shaken for 12 h at room temperature. It is noteworthy to mention that to avoid total evaporation of DCM during the loading step, the argon inlet was removed after 30 min of reaction. The resin was filtered and washed with DCM (3×), MeOH (3×), and again with DCM, then dried overnight under a vacuum to afford 3.34 g of resin **19** globally. The loading yield, calculated by the mass increase, was 70%. The deprotection step was also run in two 25 mL peptide flasks using the same amount of resin. Resin **19** (1.67 g) was swollen in 15 mL of a solution of piperidine (20%) in DCM and shaken for 90 min at room temperature. Then, the resin was filtered and washed with DCM (3×), MeOH (3×), and again with DCM, and dried under a vacuum for 16 h. Acidic mini-cleavage (5% TFA/DCM, 10 min) of a sample of resin **20** and TLC analysis confirmed the complete deprotection of the secondary amine. The two batches of resin **20** prepared as described above were mixed before the next step. Then, 30 bottom fritted reaction vessels of the 96 solid-phase reaction block of ACT LabTech manual synthesizer were loaded with 80 mg of resin **20**, which correspond to 0.052 mmol of compound **6a** (without the Fmoc protective group).

#### 4.2.5. Introduction of two levels of molecular diversity (libraries A and B)

Five stock solutions, each containing 2 equivalents (6×) of Fmoc-protected amino acid [Fmoc-*L*-Phe-OH (259 mg, 0.672 mmol), Fmoc-*L*-Ile-OH (236 mg, 0.672 mmol), Fmoc-*L*-Pro-OH (226 mg, 0.672 mmol), Fmoc-*L*-Ala-OH (208 mg, 0.672 mmol) or Fmoc-Gly-OH (199 mg, 0.672 mmol)], 2 equivalents (6×) of benzotriazole-yl-oxy-tris-pyrrolidinophosphonium hexafluorophosphate (PyBOP) (348 mg, 0.672 mmol) and 2 equivalents (6×) of hydroxybenzotriazole (HOBt) (91 mg, 0.672 mmol), were prepared in dry DMF (5.0 mL). Prior to addition to the resin, 4 equivalents (6×) of DIPEA (230 µL, 1.34 mmol) were added to each stock solution. To each of the 30 resins **20** was added 0.8 mL of the appropriate stock solution. The resins were shaken under argon for 3 h at room temperature, then filtered, washed with DMF (2×), MeOH (3×) and DCM (3×) and dried under a vacuum. TLC analysis after a mini-cleavage test (5% TFA/DCM, 10 min) with samples of resins **21** revealed that the coupling reactions were not completed. Thus, the coupling step was repeated using the same procedure as described above. This time, TLC analysis after a mini-cleavage with sample of resins **21** indicated the completion of the coupling reaction. Thus, the resins **21** were reacted 1 h with 1.0 mL of a solution of piperidine (20%) in DCM to remove the Fmoc protective group. The resins were then filtered, washed with DCM (3×), MeOH (3×) and DCM (1×), and dried under a vacuum to give 5 groups of resin of general structure **22**. The second level of molecular diversity was introduced by a similar coupling procedure described for the introduction of the first level. Thus, 6 stock solutions, each containing 2 equivalents (5x) of carboxylic acid building blocks [4-pyridyl-acetic acid (89 mg, 0.515 mmol), benzoic acid (63 mg, 0.515 mmol), **14** (192 mg, 0.515 mmol), 2-pyrazinecarboxylic acid (64 mg, 0.515 mmol), 3-aminopyrazine-2-carboxylic acid (72 mg, 0.515 mmol) or indole-2-carboxylic acid (83 mg, 0.515 mmol)], 2 equivalents (5x) of bromo-tris-pyrrolidino-phosphonium hexafluorophosphate (PyBrOP) (240 mg, 0.515 mmol) and 2 equivalents (5x) of HOBt (70 mg, 0.515 mmol), were prepared in 4 mL of dry DMF and 4 equivalents (5x) of DIPEA (180 µL, 1.03 mmol) was added. To each of the 30 resins **22** was added 0.8 mL of the appropriate stock solution. The resins were shaken under an argon atmosphere for 3 h at room temperature, then filtered, washed with MeOH (1×), DMF (2×), MeOH (3×) and DCM (2×) and dried under a vacuum. The completion of the reaction was monitored by TLC analysis of acidic mini-cleavage of samples of resins **25** (when Rj contains a Fmoc protecting group) and **27** (when Rj contains any Fmoc protecting group). As for the introduction of the first level of molecular diversity, the acylation step was repeated (2 times) as described above to afford completion.

#### 4.2.6. Removal of protective groups (libraries A and B)

Resins **25**, containing the carboxylic acid **14** as building block were treated with 1 mL of a solution of piperidine (20%) in DCM to remove the Fmoc protective group. The mixtures were shaken for 1 h at room temperature and then filtered, washed with DCM (2×) and MeOH (2×), and dried under a vacuum. Completion of the reactions was confirmed by TLC analysis after a sampling mini-cleavage (5% TFA/DCM, 10 min) of resins **26**. The acetate protective group of resins **26** or **27** was removed with 1 mL of a solution of 1 M MeONa in MeOH (25%) in THF. The mixtures were shaken for 48 h at room temperature and then, filtered, washed with MeOH (3×) and DCM (2×), and dried under a vacuum. TLC analysis of acidic mini-cleavage of sampling of resins **28** confirmed the complete deprotection of C17β-alcohol.

#### 4.2.7. Generation of E_2_ derivatives (library B) by nucleophilic cleavage

To each of the 30 resins **28** was added a solution of diethylamine (DEA, 30% in THF, 1.5 mL). The mixtures were shaken for 24 h at room temperature, then filtered and washed with acetone (3×). The filtrates were collected in preweighed tubes and evaporated in a Speedvac apparatus. Each product was dissolved in EtOAc, evaporated twice, and dried under a vacuum pump in order to obtain the DEA-free product. The phenol derivatives **59–88** (2.6–18.8 mg, 10–63% overall yield from **19**, Table 2) were obtained in high average HPLC purity (90%) according to a random sampling of 6 library members, compounds **61**, **69**, **74**, **78**, **82** and **83**.

*16β-(N-4-Amino-phenylacetyl-L-phenylalanine-aminopropyl)-3,17β-dihydroxy-estra-1,3,5(10)-triene* (**61**). Yellowish solid (8.2 mg, 26% yield); IR (film) 3292 (OH, NH and NH_2_), 1641 (C=O, amide); ^1^H- NMR (400 MHz, methanol-d_4_) δ 0.78 (s, 18-CH_3_), 0.80 to 2.35 (m, CH and CH_2_ of steroid skeleton and alkyl chain), 2.77 (m, 6-CH_2_), 2.91 and 3.05 (2m, CHCH_2_Ph), 3.13 (t, *J* = 6.5 Hz, CH_2_NH), 3.37 (s, CH_2_PhNH_2_), 3.70 (d, *J* = 9.9 Hz, 17α-CH), 4.55 (m, COCHNH), 6.49 (d, *J* = 2.5 Hz, 4-CH), 6.55 (dd, *J_1_* = 8.4 Hz, *J_2_* = 2.6 Hz, 2-CH), 6.65 (d, *J* = 8.4 Hz, 2 × CH of aminophenyl), 6.90 (d, *J* = 8.4 Hz, 2 × CH of aminophenyl), 7.08 (d, *J* = 8.4 Hz, 1-CH), 7.20 (m, 5 × CH of phenyl); LRMS calculated for C_38_H_48_N_3_O_4_ [M+H]^+^ 610.4, found 610.4 m/z; HPLC purity = 85%.

*16β-(N-3-Amino-2-pyrazinoyl-L-isoleucine-aminopropyl)-3,17β-dihydroxy-estra-1,3,5(10)-triene* (**69**). Light yellow solid (6.4 mg, 22% yield); IR (film) 3312 (OH, NH and NH_2_), 1649 (C=O, amide); ^1^H-NMR (400 MHz, methanol-d_4_) δ 0.74 (s, 18-CH_3_), 0.90 to 2.30 (m, CH and CH_2_ of steroid skeleton and alkyl chain), 0.96 (t, *J* = 7.4 Hz, CH_2_CH_3_), 1.00 (d, *J* = 6.8 Hz, CHCH_3_), 2.77 (m, 6-CH_2_), 3.24 (m, CH_2_NH and CHCH_3_), 3.69 (d, *J* = 9.9 Hz, 17α-CH), 4.39 (d, *J* = 7.6 Hz, COCHNH), 6.49 (d, *J* = 2.6 Hz, 4-CH), 6.55 (dd, *J_1_* = 8.4 Hz, *J_2_* = 2.6 Hz, 2-CH), 7.08 (d, *J* = 8.5 Hz, 1-CH), 7.84 (d, *J* = 2.4 Hz, 1 × CH of pyrazine), 8.10 (d, *J* = 2.4 Hz, 1 × CH of pyrazine); LRMS calculated for C_32_H_46_N_5_O_4_ [M+H]^+^ 564.4, found 564.5 m/z; HPLC purity = 95%.

*16β-(N-2-Pyrazinoyl-L-proline-aminopropyl)-3,17β-dihydroxy-estra-1,3,5(10)-triene* (**74**). Yellowish solid (11.7 mg, 43% yield); IR (film) 3312 (OH and NH), 1631 (C=O, amide); ^1^H-NMR (400 MHz, methanol-d_4_) δ 0.70 to 2.40 (m, CH and CH_2_ of steroid skeleton and alkyl chain), 0.78 and 0.79 (2s, 18-CH_3_), 2.79 (m, 6-CH_2_), 3.05 and 3.23 (2m, CH_2_NH), 3.71 to 3.95 (m, CH_2_N of proline and 17α-CH), 4.60 and 5.05 (2m, CHN of proline), 6.49 (s, 4-CH), 6.55 (dd, *J_1_* = 8.4 Hz, *J_2_* = 2.6 Hz, 2-CH), 7.08 (d, *J* = 8.5 Hz, 1-CH), 8.69 (m, 2 × CH of pyrazine), 9.05 (dd, *J_1_* = 2.5 Hz, *J_2_* = 1.5 Hz, 1 × CH of pyrazine); LRMS calculated for C_31_H_41_N_4_O_4_ [M+H]^+^ 533.3, found 533.4 m/z; HPLC purity = 91%.

*16β-(N-Benzoyl-L-alanine-aminopropyl)-3,17β-dihydroxy-estra-1,3,5(10)-triene* (**78**). Light yellow solid (5.4 mg, 21% yield). HPLC purity = 90% [50% of MeOH/H_2_O (90:10) and 50% of H_2_O, both containing 20 mM of NH_4_OAc]. The characterization of compound **78** was reported earlier by us [30].

*16β-(N-1H-Indole-2-carbonyl-L-alanine-aminopropyl)-3,17β-dihydroxy-estra-1,3,5(10)-triene* (**82**). Light yellow solid (3.7 mg, 13% yield); IR (film) 3292 (OH and NH), 1650 (C=O, amide); ^1^H -NMR (400 MHz, methanol-d_4_) δ 0.72 (s, 18-CH_3_), 0.85 to 2.30 (m, CH and CH_2_ of steroid skeleton and alkyl chain), 1.50 (d, *J* = 7.2 Hz, CHCH_3_), 2.75 (m, 6-CH_2_), 3.22 (m, CH_2_NH), 3.69 (d, *J* = 9.9 Hz, 17α-CH), 4.59 (q, *J* = 7.2 Hz, CHCH_3_), 6.48 (d, *J* = 2.6 Hz, 4-CH), 6.55 (d, *J* = 8.4 Hz, 2-CH), 7.06 (m, 1-CH and 1 × CH of indole), 7.20 (m, 2 × CH of indole), 7.43 (dd, *J_1_* = 8.3 Hz, *J_2_* = 0.8 Hz, 1 × CH of indole), 7.61 (d, *J* = 8.1 Hz, 1 × CH of indole); LRMS calculated for C_33_H_42_N_3_O_4_ [M+H]^+^ 544.3, found 544.5 m/z; HPLC purity = 89%.

*16β-(N-4-Pyridylacetyl-glycine-aminopropyl)-3,17β-dihydroxy-estra-1,3,5(10)-triene* (**83**). White solid (7.3 mg, 28% yield); IR (film) 3284 (OH and NH), 1649 (C=O, amide); ^1^H-NMR (400 MHz, methanol-d_4_) δ 0.79 (s, 18-CH_3_), 0.80 to 2.30 (m, CH and CH_2_ of steroid skeleton and alkyl chain), 2.79 (m, 6-CH_2_), 3.23 (m, CH_2_NH), 3.69 (s, CH_2_pyridine), 3.71 (d, *J* = 10.0 Hz, 17α-CH), 3.86 (s, COCH_2_NH), 6.49 (d, *J* = 2.5 Hz, 4-CH), 6.55 (dd, *J_1_* = 8.4 Hz, *J_2_* = 2.6 Hz, 2-CH), 7.09 (d, *J* = 8.6 Hz, 1-CH), 7.43 (dd, *J_1_* = 4.6 Hz, *J_2_* = 1.4 Hz, 2′-CH and 6′-CH of pyridine), 8.48 (dd, *J_1_* = 4.6 Hz, *J_2_* = 1.6 Hz, 3′-CH and 5′-CH of pyridine); LRMS calculated for C_30_H_40_N_3_O_4_ [M+H]^+^ 506.3, found 506.5 m/z; HPLC purity = 88%.

#### 4.2.8. Generation of sulfamoylated E_2_ derivatives (library A) by acidic cleavage

To each of the 30 resins **28** which have already been treated by nucleophilic cleavage conditions, was added 1 mL of a solution of 1,1,1,3,3,3-hexafluoro-2-propanol (HFIP) (30%) in DCM. The mixtures were shaken for 6 h at room temperature, then filtered and washed with acetone (2×). The filtrates were collected in preweighed tubes, evaporated in a Speedvac apparatus and dried under a vacuum pump. The sulfamate derivatives **29–58** (10.5–18.2 mg, 35–53% overall yield from **19**, Table 1) were obtained in high average HPLC purity (90%) according to a random sampling of six library members, compounds **30**, **38**, **41**, **49**, **51** and **58**.

*3-O-Sulfamate-16β-(N-benzoyl-L-phenylalanine-aminopropyl)-17β-hydroxy-estra-1,3,5(10)-triene* (**30**). Light yellow solid (15.4 mg, 45% yield); IR (film) 3307 (OH, NH and NH_2_), 1636 (C=O, amide), 1374 and 1184 (S=O, sulfamate); ^1^H-NMR (400 MHz, methanol-d_4_) δ 0.77 (s, 18-CH_3_), 0.95 to 2.40 (m, CH and CH_2_ of steroid skeleton and alkyl chain), 2.88 (m, 6-CH_2_), 3.05 and 3.19 (2m, CHCH_2_Ph and CH_2_NH), 3.72 (d, *J* = 9.8 Hz, 17α-CH), 4.80 (m, COCHNH), 7.02 (d, *J* = 2.4 Hz, 4-CH), 7.06 (dd, *J_1_* = 8.4 Hz, *J_2_* = 2.6 Hz, 2-CH), 7.20 to 7.55 (m, 1-CH, 5 × CH of phenyl, and 3′-CH, 4′-CH and 5′-CH of benzoyl), 7.76 (dd, *J_1_* = 8.5 Hz, *J_2_* = 1.4 Hz, 2′-CH and 6′-CH of benzoyl); LRMS calculated for C_37_H_46_N_3_O_6_S [M+H]^+^ 660.3, found 660.4 m/z; HPLC purity = 89%.

*3-O-Sulfamate-16β-(N-2-pyrazinoyl-L-isoleucine-aminopropyl)-17β-hydroxy-estra-1,3,5(10)-triene* (**38**). Light yellow solid (16.2 mg, 50% yield). HPLC purity = 90%. The characterization of compound **38** was reported earlier by us [30].

*3-O-Sulfamate-16β-(N-4-pyridylacetyl-L-proline-aminopropyl)-17β-hydroxy-estra-1,3,5(10)-triene* (**41**). Light yellow solid (15.0 mg, 47% yield); IR (film) 3292 (OH, NH and NH_2_), 1641 (C=O, amide), 1374 and 1184 (S=O, sulfamate); ^1^H-NMR (400 MHz, methanol-d_4_) δ 0.78 (s, 18-CH_3_), 0.80 to 2.40 (m, CH and CH_2_ of steroid skeleton and alkyl chain), 2.89 (m, 6-CH_2_), 3.22 (t, *J* = 7.1 Hz, CH_2_NH), 3.33 (COCH_2_pyridine under solvent peaks), 3.70 (m, 17α-CH and CH_2_N of proline), 4.40 (m, CHN of proline), 7.03 (d, *J* = 2.4 Hz, 4-CH), 7.06 (dd, *J_1_* = 8.5 Hz, *J_2_* = 2.5 Hz, 2-CH), 7.34 (d, *J* = 6.6 Hz, 1-CH), 7.40 (dd, *J_1_* = 4.6 Hz, *J_2_* = 1.6 Hz, 2′-CH and 6′-CH of pyridine), 8.47 (dd, *J_1_* = 4.5 Hz, *J_2_* = 1.6 Hz, 3′-CH and 5′-CH of pyridine); LRMS calculated for C_33_H_45_N_4_O_6_S [M+H]^+^ 625.3, found 625.4 m/z; HPLC purity = 89%.

*3-O-Sulfamate-16β-(N-4-amino-phenylacetyl-L-alanine-aminopropyl)-17β-hydroxy-estra-1,3,5(10)-triene* (**49**). Light yellow solid (12.1 mg, 38% yield); IR (film) 3308 (OH, NH and NH_2_), 1652 (C=O, amide), 1374 and 1184 (S=O, sulfamate); ^1^H-NMR (400 MHz, methanol-d_4_) δ 0.79 (s, 18-CH_3_), 0.90 to 2.40 (m, CH and CH_2_ of steroid skeleton and alkyl chain), 1.33 (d, *J* = 7.1 Hz, CHCH_3_), 2.89 (m, 6-CH_2_), 3.18 (t, *J* = 7.1 Hz, CH_2_NH), 3.43 (s, CH_2_PhNH_2_), 3.73 (d, *J* = 9.8 Hz, 17α-CH), 4.30 (q, *J* = 7.2 Hz, CHCH_3_), 6.70 (dd, *J_1_* = 6.4 Hz, *J_2_* = 2.0 Hz, 2 × CH of aminophenyl), 7.04 (m, 2-CH, 4-CH and 2 × CH of aminophenyl), 7.35 (d, *J* = 8.6 Hz, 1-CH); LRMS calculated for C_32_H_45_N_4_O_6_S [M+H]^+^ 613.3, found 613.3 m/z; HPLC purity = 91%.

*3-O-Sulfamate-16β-(N-3-amino-pyrazinoyl-L-alanine-aminopropyl)-17β-hydroxy-estra-1,3,5(10)-triene* (**51**). Light yellow solid (14.5 mg, 47% yield); IR (film) 3346 (OH, NH and NH_2_), 1646 (C=O, amide), 1375 and 1186 (S=O, sulfamate); ^1^H-NMR (400 MHz, methanol-d_4_) δ 0.77 (s, 18-CH_3_), 0.95 to 2.40 (m, CH and CH_2_ of steroid skeleton and alkyl chain), 1.47 (d, *J* = 7.1 Hz, CHCH_3_), 2.88 (m, 6-CH_2_), 3.23 (m, CH_2_NH), 3.72 (d, *J* = 9.8 Hz, 17α-CH), 4.54 (q, *J* = 7.1 Hz, CHCH_3_), 7.02 (d, *J* = 2.4 Hz, 4-CH), 7.06 (dd, *J_1_* = 8.6 Hz, *J_2_* = 2.5 Hz, 2-CH), 7.33 (d, *J* = 8.6 Hz, 1-CH), 7.86 (d, *J* = 2.4 Hz, 1 × CH of pyrazine), 8.13 (d, *J* = 2.4 Hz, 1 × CH of pyrazine); LRMS calculated for C_29_H_41_N_6_O_6_S [M+H]^+^ 601.3, found 601.3 m/z; HPLC purity = 90%.

*3-O-Sulfamate-16β-(N-1H-indole-2-carbonyl-glycine-aminopropyl)-17β-hydroxy-estra-1,3,5(10)-triene* **58**). White solid (13.0 mg, 41% yield); IR (film) 3292 (OH, NH and NH_2_), 1636 (C=O, amide), 1374 and 1186 (S=O, sulfamate); ^1^H-NMR (400 MHz, methanol-d_4_) δ 0.75 (s, 18-CH_3_), 0.95 to 2.35 (m, CH and CH_2_ of steroid skeleton and alkyl chain), 2.88 (m, 6-CH_2_), 3.26 (m, CH_2_NH), 3.71 (d, *J* = 9.9 Hz, 17α-CH), 4.05 (s, COCH_2_NH), 7.02 (d, *J* = 2.5 Hz, 4-CH), 7.06 (m, 2-CH and 1 × CH of indole), 7.14 (d, *J* = 0.8 Hz, 1 × CH of indole), 7.22 (dt, *J_1_* = 8.0 Hz, *J_2_* = 1.2 Hz, 1 × CH of indole), 7.33 (d, *J* = 8.4 Hz, 1-CH), 7.44 (dd, *J_1_* = 8.3 Hz, *J_2_* = 0.8 Hz, 1 × CH of indole), 7.62 (d, *J* = 8.1 Hz, 1 × CH of indole); LRMS calculated for C_32_H_41_N_4_O_6_S [M+H]^+^ 609.3, found 609.2 m/z; HPLC purity = 89%.

#### 4.2.9. Synthesis of resin **20** for preparation of library C

The loading procedure described above for the preparation of libraries A and B was used without any significant modifications. Briefly, trityl chloride resin (10.1 g, 1.50 mmol/g theoretical loading) and precursor **6a** (5.11 g, 7.6 mmol) were put in a round 250 mL dry flask equipped with a septum and an argon inlet. DCM (100 mL) was added and the reaction mixture was stirred for 3 min. Then, DIPEA (24.3 mL) was added and the mixture was shaken at room temperature. After 30 min of reaction, the argon inlet was removed. After 16 h, the mixture was filtered, washed with DCM (3×) and MeOH (3×), and dried under a vacuum to afford 14.1 g of resin **19**. The loading yield, calculated by the mass increase, was 42%. It is noteworthy to mention that the loading could not be higher than 50% because 2 equivalents of trityl chloride resin were used for 1 equivalent of precursor **6a**. Then 63 fritted bottom reaction vessels of the 96 solid-phase reaction block of ACT LabTech manual synthesizer were loaded with 200 mg of resin **19**. A solution of piperidine (20%) in DCM was added to resins **19** and the mixtures were shaken for 1 h at room temperature to remove the Fmoc protective group. Then, the resin was filtered and washed with DCM (2×), MeOH (3×), and dried under a vacuum for 16 h. The completion of the deprotection was proven by acidic mini-cleavage as described above. Thus, each reaction vessel contains resins loading 0.109 mmol of compound **6a** (without the Fmoc protective group).

#### 4.2.10. Introduction of three levels of molecular diversity (library C)

The levels of molecular diversity were introduced using a similar procedure as the one described above for the preparation of libraries A and B. To introduce the first level of molecular diversity, three stock solutions, each containing 2 equivalents (21×) of Fmoc-protected amino acid [Fmoc-Gly-OH (1.36 g, 4.56 mmol), Fmoc-*L*-Phe-OH (1.77 g, 4.56 mmol) or Fmoc-*L*-4-NO_2_-Phe-OH (1.98 g, 4.56 mmol)], 2 equivalents (21×) of PyBOP (2.37 g, 4.56 mmol) and 2 equivalents (21×) of HOBt (616 mg, 4.56 mmol), were prepared in dry DMF (42 mL). Prior to addition to the resin, 4 equivalents (21×) of DIPEA (1.59 mL, 9.12 mmol) were added to each stock solution. To each of the 63 resins **20** was added 2.0 mL of the appropriate stock solution. The resins were shaken under an argon atmosphere for 2 h at room temperature, then filtered, washed with DMF (2×), DCM (2×) and MeOH (3×) and dried under a vacuum. This procedure was repeated (3 times) until completion of the reaction was afforded and verified by acidic mini-cleavage as described above. The cleavage of Fmoc group of resins **21** was then performed by a 1 h treatment with 2.0 mL of a solution of piperidine (20%) in DCM. The resins were then filtered, washed with DCM (3×), MeOH (3×), and dried under a vacuum to give three groups of resins of general structure **22**. The second level of molecular diversity was introduced using the same building blocks and the same procedure described above for the introduction of the first level of molecular diversity. This time, the coupling procedure was repeated twice to afford completion of the reaction by TLC analysis after acidic mini-cleavage. Resins of general structure **23** were then treated with a solution of piperidine (20%) in DCM for 1 h at room temperature. The resins were filtered, washed with DCM (2×) and MeOH (2×), and dried to afford resins of general structure **24**. The third level of molecular diversity was introduced by coupling resins **24** with carboxylic acid building blocks **12–18**. Seven stock solutions, each containing 2 equivalents (9x) of carboxylic acid building blocks **12–18** [**12** (701 mg, 1.95 mmol), **13** (701 mg, 1.95 mmol), **14** (729 mg, 1.95 mmol), **15** (729 mg, 1.95 mmol), **16** (756 mg, 1.95 mmol), **17** (756 mg, 1.95 mmol) or **18** (784 mg, 1.95 mmol)], 2 equivalents (9x) of PyBrOP (911 mg, 1.95 mmol) and 2 equivalents of HOBt (264 mg, 1.95 mmol), were prepared in dry DMF (18 mL) and 4 equivalents (9x) of DIPEA (681 µL, 3.90 mmol) were added. To each of the 63 resins **24** was added 2.0 mL of the appropriate stock solution. The resins were shaken under an argon atmosphere for 2 h at room temperature, then filtered, washed with DMF (2×), DCM (2×) and MeOH (3×) and dried under a vacuum to afford resins of general structure **25** (n = 2). The completion of the coupling step was proven by acidic mini-cleavage and TLC analysis of samples of resins **25**.

#### 4.2.11. Removal of protective groups (library C)

The Fmoc protective group from carboxylic acid building blocks on resins **25** (n = 2) was removed with the same procedure described above for the preparation of libraries A and B. The acetate protective group of resins **26** (n = 2) was removed using a similar procedure described above for the preparation of libraries A and B. However, the mixtures were shaken for 100 h instead of 48 h in order to obtain complete deprotection of the steroidal C17β-hydroxy by TLC analysis after acidic mini-cleavage of samples of resins **28** (n = 2).

#### 4.2.12. Generation of E_2_ derivatives (library C) by nucleophilic cleavage

To each of the 63 resins **28** (n = 2) was added 1.5 mL of a solution of DEA (30%) in THF. The mixtures were shaken for 50 h at room temperature. Then 1.0 mL of a solution of DEA (30%) in THF was added to each of the 63 resins and the mixtures were shaken for an additional 16 h. The mixtures were next filtered and washed with THF (1×) and MeOH (3×). The filtrates were collected in preweighed tubes and evaporated in a Speedvac apparatus. Each product was dissolved in THF, evaporated and dried under a vacuum pump in order to obtain the DEA-free product. The phenol derivatives **89–151** (5.3–33.5 mg, 6–41% overall yield from **19**, Table 3) were obtained in purity around 70 to 75% according to TLC analysis of all library members and ^1^H-NMR and LRMS analysis of a random sampling of 14 library members, compounds **89**, **94**, **99**, **103**, **109**, **111**, **114**, **118**, **121**, **126**, **133**, **143**, **144** and **148**.

*16β-(N-4-Aminobenzoyl-glycine-glycine-aminopropyl)-3,17β-dihydroxy-estra-1,3,5(10)-triene* (**89**). Yellowish solid (16.4 mg, 27% yield); ^1^H-NMR (400 MHz, methanol-d_4_) δ 0.78 (s, 18-CH_3_), 0.85 to 2.35 (m, CH and CH_2_ of steroid skeleton and alkyl chain), 2.78 (m, 6-CH_2_), 3.23 (m, CH_2_NH), 3.69 (d, *J* = 10.4 Hz, 17α-CH), 3.86 and 3.99 (2m, 2 × COCH_2_NH), 6.49 (d, *J* = 2.2 Hz, 4-CH), 6.55 (dd, *J_1_* = 8.6 Hz, *J_2_* = 2.5 Hz, 2-CH), 6.68 (d, *J* = 7.0 Hz, 3′-CH and 5′-CH of 4-aminobenzoyl), 7.09 (d, *J* = 8.4 Hz, 1-CH), 7.69 (dd, *J_1_* = 8.5 Hz, *J_2_* = 1.4 Hz, 2′-CH and 6′-CH of 4-aminobenzoyl); LRMS calculated for C_32_H_43_N_4_O_5_ [M+H]^+^ 563.3, found 563.3 m/z.

*16β-(N-3-Aminophenyl-propanoyl-glycine-glycine-aminopropyl)-3,17β-dihydroxy-estra-1,3,5(10)-triene* (**94**). Yellowish solid (16.5 mg, 26% yield); ^1^H-NMR (400 MHz, methanol-d_4_) δ 0.79 and 0.80 (2s, 18-CH_3_), 0.85 to 2.35 (m, CH and CH_2_ of steroid skeleton and alkyl chain), 2.58 (t, *J* = 7.6 Hz, COCH_2_CH_2_), 2.78 (m, 6-CH_2_), 2.84 (m, COCH_2_CH_2_), 3.25 (m, CH_2_NH), 3.75 (under THF peaks, 17α-CH), 3.83 (s, 2 × COCH_2_NH), 6.56 (m, 2-CH, 4-CH and 3 × CH of 3-aminophenyl), 7.06 (m, 1-CH and 1 × CH of 3-aminophenyl); LRMS calculated for C_32_H_43_N_4_O_5_ [M-H]^-^ 589.3, found 589.5 m/z.

*16β-(N-3-Aminophenylacetyl-L-phenylalanine-glycine-aminopropyl)-3,17β-dihydroxy-estra-1,3,5(10)-triene* (**99**). Yellowish solid (20.4 mg, 28% yield); ^1^H-NMR (400 MHz, methanol-d_4_) δ 0.79 (s, 18-CH_3_), 0.85 to 2.35 (m, CH and CH_2_ of steroid skeleton and alkyl chain), 2.78 (m, 6-CH_2_), 2.98 and 3.17 (2m, CHCH_2_Ph and CH_2_NH), 3.43 (s, COCH_2_PhNH_2_), 3.75 (under THF peaks, 17α-CH), 3.92 (m, COCH_2_NH), 4.46 (m, CHCH_2_Ph), 6.58 (m, 2-CH, 4-CH and 3 × CH of 3-aminophenyl), 7.16 (m, 1-CH, 1 × CH of 3-aminophenyl, 5 × CH of phenyl); LRMS calculated for C_40_H_49_N_4_O_5_ [M-H]^-^ 665.4, found 665.5 m/z.

*16β-(N-4-Aminobenzoyl-L-4-nitro-phenylalanine-glycine-aminopropyl)-3,17β-dihydroxy-estra-1,3,5(10)-triene* (**103**). Yellowish solid (13.9 mg, 18% yield); ^1^H-NMR (400 MHz, methanol-d_4_) δ 0.78 (m, 18-CH_3_), 0.80 to 2.40 (m, CH and CH_2_ of steroid skeleton and alkyl chain), 2.78 (m, 6-CH_2_), 3.24 (m, CH_2_NH and CHCH_2_PhNO_2_), 3.75 (under THF peaks, 17α-CH), 3.92 (m, COCH_2_NH), 4.68 (m, CHCH_2_PhNO_2_), 6.49 (s, 4-CH), 6.55 (dd, *J_1_* = 8.4 Hz, *J_2_* = 0.8 Hz, 2-CH), 6.65 (dd, *J_1_* = 8.8 Hz, *J_2_* = 2.2 Hz, 3′-CH and 5′-CH of 4-aminobenzoyl), 7.09 (d, *J* = 8.4 Hz, 1-CH), 7.54 (d, *J* = 8.6 Hz, 2′-CH and 6′-CH of 4-nitro-phenyl), 7.58 (m, 2′-CH and 6′-CH of 4-aminobenzoyl), 8.18 (m, 3′-CH and 5′-CH of 4-nitro-phenyl); LRMS calculated for C_39_H_48_N_5_O_7_ [M+H]^+^ 698.3, found 698.2 m/z.

*16β-(N-4-aminophenyl-butanoyl-L-4-nitro-phenylalanine-glycine-aminopropyl)-3,17β-dihydroxy-estra-1,3,5(10)-triene* (**109**). Yellowish solid (25.5 mg, 32% yield); ^1^H-NMR (400 MHz, methanol-d_4_) δ 0.78 (s, 18-CH_3_), 0.80 to 2.35 (m, CH and CH_2_ of steroid skeleton and alkyl chain), 2.41 (m, COCH_2_CH_2_), 2.54 (t, *J* = 7.8 Hz, CH_2_PhNH_2_), 2.78 (m, 6-CH_2_), 3.21 (m, CH_2_NH and CHCH_2_PhNO_2_), 3.75 (under THF peaks, 17α-CH), 3.91 (m, COCH_2_NH), 4.59 (m, CHCH_2_PhNO_2_), 6.49 (s, 4-CH), 6.55 (d, *J* = 8.4 Hz, 2-CH), 6.67 (m, 3′-CH and 5′-CH of 4-aminophenyl), 6.92 (m, 2′-CH and 6′-CH of 4-aminophenyl), 7.09 (d, *J* = 8.3 Hz, 1-CH), 7.51 (d, *J* = 8.6 Hz, 2′-CH and 6′-CH of 4-nitro-phenyl), 8.17 (d, *J* = 8.6 Hz, 3′-CH and 5′-CH of 4-nitro-phenyl); LRMS calculated for C_42_H_54_N_5_O_7_ [M+H]^+^ 740.4, found 740.5 m/z.

*16β-(N-3-Aminobenzoyl-glycine-L-phenylalanine-aminopropyl)-3,17β-dihydroxy-estra-1,3,5(10)-triene* (**111**). Yellowish solid (28.5 mg, 40% yield); ^1^H-NMR (400 MHz, methanol-d_4_) δ 0.78 (s, 18-CH_3_), 0.85 to 2.35 (m, CH and CH_2_ of steroid skeleton and alkyl chain), 2.78 (m, 6-CH_2_), 2.95 and 3.16 (2m, CHCH_2_Ph and CH_2_NH), 3.75 (under THF peaks, 17α-CH), 3.95 (m, COCH_2_NH), 4.60 (m, CHCH_2_Ph), 6.49 (s, 4-CH), 6.55 (dd, *J_1_* = 8.5 Hz, *J_2_* = 2.4 Hz, 2-CH), 6.88 (d, *J* = 7.6 Hz, 4′-CH of 3-aminophenyl), 7.05 to 7.35 (m, 1-CH, 2′-CH, 5′-CH and 6′-CH of 3-aminophenyl and 5 × CH of phenyl); LRMS calculated for C_39_H_49_N_4_O_5_ [M+H]^+^ 653.4, found 653.2 m/z.

*16β-(N-4-Aminophenyl-propanoyl-glycine-L-phenylalanine-aminopropyl)-3,17β-dihydroxy-estra-1,3,5(10)-triene* (**114**). Yellowish solid (25.6 mg, 35% yield); ^1^H-NMR (400 MHz, methanol-d_4_) δ 0.78 (s, 18-CH_3_), 0.85 to 2.40 (m, CH and CH_2_ of steroid skeleton and alkyl chain), 2.47 (m, CH_2_CH_2_CO), 2.79 (m, 6-CH_2_ and CH_2_CH_2_CO), 2.92 and 3.15 (2m, CHCH_2_Ph and CH_2_NH), 3.75 (under THF peaks, 17α-CH), 3.93 (m, COCH_2_NH), 4.57 (m, CHCH_2_Ph), 6.49 (s, 4-CH), 6.55 (dd, *J_1_* = 8.4 Hz, *J_2_* = 2.4 Hz, 2-CH), 6.67 (d, *J* = 8.3 Hz, 3′-CH and 5′-CH of 4-aminophenyl), 6.97 (d, *J* = 8.3 Hz, 2′-CH and 6′-CH of 4-aminophenyl), 7.09 (d, *J* = 8.4 Hz, 1-CH), 7.25 (m, 5 × CH of phenyl); LRMS calculated for C_41_H_53_N_4_O_5_ [M+H]^+^ 681.4, found 681.5 m/z.

*16β-(N-3-Aminobenzoyl-L-phenylalanine-L-phenylalanine-aminopropyl)-3,17β-dihydroxy-estra-1,3,5(10)-triene* (**118**). Yellowish solid (32.9 mg, 41% yield); ^1^H-NMR (400 MHz, methanol-d_4_) δ 0.79 (s, 18-CH_3_), 0.85 to 2.35 (m, CH and CH_2_ of steroid skeleton and alkyl chain), 2.78 (m, 6-CH_2_), 3.00 and 3.14 (2m, 2 × CHCH_2_Ph and CH_2_NH), 3.75 (under THF peaks, 17α-CH), 4.56 and 4.77 (2m, 2 × CHCH_2_Ph), 6.48 (d, *J* = 2.4 Hz, 4-CH), 6.55 (dd, *J_1_* = 8.4 Hz, *J_2_* = 2.6 Hz, 2-CH), 6.86 (d, *J* = 7.9 Hz, 4′-CH of 3-aminophenyl), 7.13 (m, 1-CH, 2′-CH, 5′-CH and 6′-CH of 3-aminophenyl and 10 × CH of phenyl); LRMS calculated for C_46_H_55_N_4_O_5_ [M+H]^+^ 743.4, found 743.2 m/z.

*16β-(N-4-Aminophenyl-propanoyl-L-phenylalanine-L-phenylalanine-aminopropyl)-3,17β-dihydroxy-estra-1,3,5(10)-triene* (**121**). Yellowish solid (29.5 mg, 35% yield); ^1^H-NMR (400 MHz, methanol-d_4_) δ 0.78 (s, 18-CH_3_), 0.85 to 2.40 (m, CH and CH_2_ of steroid skeleton and alkyl chain), 2.65 (t, *J* = 7.8 Hz, CH_2_CO), 2.78 (m, 6-CH_2_ and CH_2_CH_2_CO), 2.93 and 3.11 (2m, 2 × CHCH_2_Ph and CH_2_NH), 3.75 (under THF peaks, 17α-CH), 4.54 (m, 2 × CHCH_2_Ph), 6.48 (s, 4-CH), 6.55 (dd, *J_1_* = 8.4 Hz, *J_2_* = 2.5 Hz, 2-CH), 6.65 (d, *J* = 8.3 Hz, 3′-CH and 5′-CH of 4-aminophenyl), 6.90 (d, *J* = 8.2 Hz, 2′-CH and 6′-CH of 4-aminophenyl), 7.09 (d, *J* = 8.4 Hz, 1-CH), 7.22 (m, 10 × CH of phenyl); LRMS calculated for C_48_H_59_N_4_O_5_ [M+H]^+^ 771.4, found 771.6 m/z.

*16β-(N-4-Aminophenyl-acetyl-L-4-nitro-phenylalanine-L-phenylalanine-aminopropyl)-3,17β-dihydroxy-estra-1,3,5(10)-triene* (**126**). Yellowish solid (27.6 mg, 32% yield); ^1^H-NMR (400 MHz, methanol-d_4_) δ 0.79 (s, 18-CH_3_), 0.85 to 2.35 (m, CH and CH_2_ of steroid skeleton and alkyl chain), 2.78 (m, 6-CH_2_), 2.94 and 3.14 (2m, CHCH_2_Ph, CHCH_2_PhNO_2_ and CH_2_NH), 3.33 (under MeOH peaks, COCH_2_PhNH_2_), 3.75 (under THF peaks, 17α-CH), 4.53 and 4.66 (2m, CHCH_2_Ph and CHCH_2_PhNO_2_), 6.48 (d, *J* = 2.4 Hz, 4-CH), 6.55 (dd, *J_1_* = 8.4 Hz, *J_2_* = 2.2 Hz, 2-CH), 6.62 (d, *J* = 8.3 Hz, 3′-CH and 5′-CH of 4-aminophenyl), 6.88 (d, *J* = 8.4 Hz, 2′-CH and 6′-CH of 4-aminophenyl), 7.09 (d, *J* = 8.5 Hz, 1-CH), 7.23 (m, 2′-CH and 6′-CH of 4-nitro-phenyl and 5 × CH of phenyl), 8.03 (d, *J* = 8.7 Hz, 3′-CH and 5′-CH of 4-nitro-phenyl); LRMS calculated for C_47_H_56_N_5_O_7_ [M+H]^+^ 802.4, found 802.4 m/z.

*16β-(N-4-Aminophenyl-acetyl-glycine-L-4-nitro-phenylalanine-aminopropyl)-3,17β-dihydroxy-estra-1,3,5(10)-triene* (**133**). Yellowish solid (20.1 mg, 26% yield); ^1^H-NMR (400 MHz, methanol-d_4_) δ 0.78 (m, 18-CH_3_), 0.85 to 2.35 (m, CH and CH_2_ of steroid skeleton and alkyl chain), 2.79 (m, 6-CH_2_), 3.01 and 3.15 (2m, CHCH_2_PhNO_2_ and CH_2_NH), 3.45 (m, COCH_2_PhNH_2_), 3.75 (under THF peaks, 17α-CH and COCH_2_NH), 4.64 (m, CHCH_2_PhNO_2_), 6.50 (s, 4-CH), 6.55 (dd, *J_1_* = 8.3 Hz, *J_2_* = 2.5 Hz, 2-CH), 6.71 (m, 3′-CH and 5′-CH of 4-aminophenyl), 7.08 (m, 1-CH and 2′-CH and 6′-CH of 4-aminophenyl), 7.45 (d, *J* = 8.6 Hz, 2′-CH and 6′-CH of 4-nitro-phenyl), 8.17 (d, *J* = 8.7 Hz, 3′-CH and 5′-CH of 4-nitro-phenyl); LRMS calculated for C_40_H_50_N_5_O_7_ [M+H]^+^ 712.4, found 712.5 m/z.

*16β-(N-3-Aminophenyl-propanoyl-L-phenylalanine-L-4-nitro-phenylalanine-aminopropyl)-3,17β-di-hydroxy-estra-1,3,5(10)-triene* (**143**). Yellowish solid (28.8 mg, 33% yield); ^1^H-NMR (400 MHz, methanol-d_4_) δ 0.79 (s, 18-CH_3_), 0.85 to 2.35 (m, CH and CH_2_ of steroid skeleton and alkyl chain), 2.46 (m, CH_2_CO), 2.76 (m, 6-CH_2_ and CH_2_CH_2_CO), 3.10 (m, CHCH_2_Ph, CHCH_2_PhNO_2_ and CH_2_NH), 3.75 (under THF peaks, 17α-CH), 4.60 (m, CHCH_2_Ph and CHCH_2_PhNO_2_), 6.55 (m, 2-CH, 4-CH, 2′-CH, 4′-CH and 6′-CH of 3-aminophenyl), 7.14 (m, 1-CH, 5′-CH of 3-aminophenyl and 5 × CH of phenyl), 7.46 (d, *J* = 8.7 Hz, 2′-CH and 6′-CH of 4-nitro-phenyl), 8.16 (d, *J* = 8.6 Hz, 3′-CH and 5′-CH of 4-nitro-phenyl); LRMS calculated for C_48_H_58_N_5_O_7_ [M+H]^+^ 816.4, found 816.4 m/z.

*16β-(N-4-Aminophenyl-butanoyl-L-phenylalanine-L-4-nitro-phenylalanine-aminopropyl)-3,17β-di-hydroxy-estra-1,3,5(10)-triene* (**144**). Yellowish solid (32.8 mg, 36% yield); ^1^H-NMR (400 MHz, methanol-d_4_) δ 0.78 (s, 18-CH_3_), 0.85 to 2.40 (m, CH and CH_2_ of steroid skeleton and alkyl chain), 2.79 (m, 6-CH_2_, CH_2_PhNH_2_), 3.12 (m, CHCH_2_Ph, CHCH_2_PhNO_2_ and CH_2_NH), 3.75 (under THP peaks, 17α-CH), 4.60 (m, CHCH_2_Ph and CHCH_2_PhNO_2_), 6.49 (s, 4-CH), 6.55 (dd, *J_1_* = 8.4 Hz, *J_2_* = 2.4 Hz, 2-CH), 6.66 (m, 3′-CH and 5′-CH of 4-aminophenyl), 6.87 (d, *J* = 8.2 Hz, 2′-CH and 6′-CH of 4-aminophenyl), 7.09 (d, *J* = 8.5 Hz, 1-CH), 7.23 (m, 5 × CH of phenyl), 7.46 (d, *J* = 8.6 Hz, 2′-CH and 6′-CH of 4-nitro-phenyl), 8.15 (d, *J* = 8.7 Hz, 3′-CH and 5′-CH of 4-nitro-phenyl); LRMS calculated for C_49_H_58_N_5_O_7_ [M-H]^-^ 828.4, found 828.3 m/z.

*16β-(N-3-aminophenyl-acetyl-L-4-nitro-phenylalanine-L-4-nitro-phenylalanine-aminopropyl)-3,17β-dihydroxy-estra-1,3,5(10)-triene* (**148**). Yellowish solid (27.4 mg, 30% yield); ^1^H-NMR (400 MHz, methanol-d_4_) δ 0.78 (s, 18-CH_3_), 0.85 to 2.40 (m, CH and CH_2_ of steroid skeleton and alkyl chain), 2.78 (m, 6-CH_2_), 3.01 and 3.16 (2m, 2 × CHCH_2_PhNO_2_, and CH_2_NH), 3.38 (m, CH_2_PhNH_2_), 3.75 (under THF peaks, 17α-CH), 4.65 (m, 2 × CHCH_2_PhNO_2_), 6.50 (m, 2-CH, 4-CH and 2′-CH, 4′-CH and 6′-CH of 3-aminophenyl), 6.96 (m, 5′-CH of 3-aminophenyl), 7.09 (d, *J* = 8.6 Hz, 1-CH), 7.39 (m, 2 × 2′-CH and 6′-CH of 4-nitro-phenyl), 8.10 (m, 2 × 3′-CH and 5′-CH of 4-nitro-phenyl); LRMS calculated for C_48_H_55_N_6_O_9_ [M+H]^+^ 847.4, found 847.3 m/z.

#### 4.2.13. Synthesis of 2-(3′-bromo-phenyl)-acetic acid tert-butyl ester (**153**)

3-Bromophenylacetic acid (**152**) (2.5 g, 11.6 mmol) was dissolved in *tert*-butanol (120 mL) under an argon atmosphere at room temperature. Then, di-*tert*-butyl dicarbonate (5.33 mL, 23.2 mmol) and DMAP (426 mg, 3.48 mmol) were added. After 24 h, the reaction mixture was evaporated under reduced pressure and the crude product was purified by flash chromatography (hexanes/EtOAc, 97:3 to 95:5) to provide ester **153** (2.95 g, 94% yield) in the form of a colourless oil. IR (film) 1734 (C=O, ester); ^1^H-NMR (400 MHz, acetone-d_6_) δ 1.44 (s, (CH_3_)_3_CO), 3.58 (s, CH_2_COO), 7.30 (s_br_, 5′-CH and 6′-CH), 7.45 and 7.51 (2s_br_, 2′-CH and 4′-CH); ^13^C-NMR (75 MHz, acetone-d_6_) δ 28.1 (3×), 42.1, 81.1, 122.5, 129.1, 130.5, 131.0, 133.1, 138.6, 170.6; LRMS calculated for C_12_H_14_BrO_2_ [M-H]^-^ 269.0, found 269.0 and 271.1 m/z.

#### 4.2.14. Synthesis of 2-(3′-vinyl-phenyl)-acetic acid tert-butyl ester (**154**)

A suspension of ester **153** (7.00 g, 25.8 mmol), tributyl(vinyl)tin (18.9 mL, 64.5 mmol), Pd_2_(dba)_3_ (2.36 g, 2.58 mmol) and P(*t*-Bu)_3_ (10% w/v in hexanes) (11.5 mL, 5.67 mmol) in anhydrous toluene (26 mL) was stirred for 16 h under argon atmosphere at room temperature. Then, diethyl ether (130 mL) and KF.2H_2_O (12.9 g) was added and the mixture was stirred for 30 min in order to quench the reaction. The suspension was filtered through a pad of celite, washed with diethyl ether, and the filtrate was evaporated to dryness. The crude product was purified by flash chromatography (hexanes/EtOAc, 97:3) to provide **154** (4.97 g, 88% yield) in the form of a colourless oil. IR (film) 1732 (C=O, ester); ^1^H-NMR (400 MHz, acetone-d_6_) δ 1.44 (s, (CH_3_)_3_CO), 3.56 (s, CH_2_COO), 5.25 (dd, *J_1_* = 10.9 Hz, *J_2_* = 0.8 Hz, CH of CH_2_=), 5.82 (dd, *J_1_* = 17.6 Hz, *J_2_* = 0.9 Hz, CH of CH_2_=), 6.76 (dd, *J_1_* = 17.6 Hz, *J_2_* = 10.9 Hz, CH=), 7.30 (m, 4 × CH of phenyl); ^13^C-NMR (100 MHz, acetone-d_6_) δ 28.1 (3×), 42.7, 80.7, 114.1, 125.3, 127.9, 129.3, 129.5, 136.1, 136.3, 137.7, 170.7.

#### 4.2.15. Synthesis of 2-[3′-(2″-hydroxy-ethyl)-phenyl]-acetic acid tert-butyl ester (**155**)

To a solution of vinyl **154** (4.89 g, 22.4 mmol) dissolved in dry THF (450 mL) at 0 °C was added 1.0 M borane-THF complex (100 mL, 100 mmol) under an argon atmosphere. The mixture was stirred at 0 °C for 3 h. Then, 4 M aqueous NaOAc solutions (28.0 mL, 112 mmol) and 30% (w/v) H_2_O_2_ (12.7 mL, 112 mmol) were added. After 2 h at 0 °C, the reaction was quenched by water and the extraction was performed with EtOAc. The organic phase was washed with brine, dried over MgSO_4_, and evaporated to dryness. The crude compound was purified by flash chromatography (hexanes/EtOAc, 7:3 to 6:4) to give alcohol **155** (2.36 g, 45% yield) in the form of a colourless oil. IR (film) 3435 (OH), 1732 (C=O, ester); ^1^H-NMR (400 MHz, acetone-d_6_) δ 1.43 (s, (CH_3_)_3_CO), 2.81 (t, *J* = 7.0 Hz, CH_2_CH_2_OH), 3.51 (s, CH_2_COO), 3.68 (m, OH), 3.75 (t, *J* = 6.8 Hz, CH_2_OH), 7.18 (m, 4 × CH of phenyl); ^13^C-NMR (75 MHz, acetone-d_6_) δ 28.0 (3×), 40.0, 42.7, 63.7, 80.5, 127.4, 128.0, 128.8, 130.5, 135.7, 140.4, 171.0; LRMS calculated for C_14_H_19_O_3_ [M-H]^-^ 235.1, found 235.1 m/z.

#### 4.2.16. Synthesis of 2-[3′-(tert-butoxycarbonylmethyl)-phenyl]-acetic acid (**156**)

Alcohol **155** (500 mg, 2.11 mmol) was dissolved in dry DCM (42 mL) under an argon atmosphere. Dess-Martin periodinane (988 mg, 2.33 mmol) was then added and the reaction was stirred at room temperature. After 1 h, the mixture was evaporated to dryness. The crude product was filtered through a pad of silica gel using hexanes/EtOAc, 9:1 as eluent to afford the aldehyde (385 mg, 78% yield) in the form of a colourless oil. ^1^H-NMR (400 MHz, acetone-d_6_) δ 1.43 (s, (CH_3_)_3_CO), 3.55 (s, CH_2_COO), 3.75 (d, *J* = 2.1 Hz, CH_2_CHO), 7.21 (m, 2′-CH, 4′-CH, 6′-CH), 7.33 (t, *J* = 7.3 Hz, 5′-CH), 9.74 (t, *J* = 2.2 Hz, CHO). The aldehyde (372 mg, 1.59 mmol) was dissolved in *t*-BuOH (22 mL) and 2-methyl-2-butene (9 mL). An oxidative solution freshly prepared by dissolving NaClO_2_ (1.59 g) and NaH_2_PO_4_ (1.59 g) in H_2_O (15.9 mL) was added and the reaction mixture was stirred for 15 min at room temperature. The reaction was quenched by addition of water and the extraction was performed with EtOAc. The organic phase was washed with brine, dried over MgSO_4_ and evaporated to dryness under reduced pressure to afford carboxylic acid **156** (350 mg, 88% yield) in the form of a colourless oil. IR (film) 3700–2300 (OH, COOH), 1731 (C=O, ester), 1713 (C=O, acide); ^1^H-NMR (400 MHz, acetone-d_6_) δ 1.44 (s, (CH_3_)_3_CO), 3.54 (s, CH_2_COO), 3.63 (s, CH_2_COOH), 7.23 (m, 4 × CH of phenyl); ^13^C-NMR (100 MHz, acetone-d_6_) δ 27.8 (3×), 40.8, 42.5, 80.4, 128.1, 128.2, 128.8, 130.7, 135.5, 135.7, 170.7, 172.4; LRMS calculated for C_14_H_17_O_4_ [M-H]^-^ 249.1, found 249.1 m/z.

#### 4.2.17. Synthesis of resin **158** for preparation of library D

Two coupling reactions were run at the same time using a 50 mL (flask A) and a 25 mL (flask B) peptide flask. A solution of precursor **6b** (2.50 g, 3.50 mmol) in dry DCM (30 mL) was prepared and 20.7 mL were added in flask A containing trityl chloride resin (3.0 g, 1.6 mmol/g theoretical loading) under an argon atmosphere. 9.3 mL of the solution of precursor **6b** in DCM was added, under argon atmosphere to flask B containing trityl chloride resin (1.36 g, 1.6 mmol/g theoretical loading). After 5 min, DIPEA (7.6 mL in flask A and 3.4 mL in flask B) was added and the mixtures were shaken for 30 min at room temperature. Then, the argon inlets were removed and the mixtures were shaken for an additional 16 h. The resin was filtered and washed with DCM (3×) and MeOH (4x), and dried overnight under a vacuum to afford resin **157**. The deprotection step was also run in 2 flasks, a 50 mL (flask A) and a 25 mL (flask B) peptide flask. Resin **157** was swollen in a solution of piperidine (20%) in DCM (25 mL in flask A and 10 mL in flask B) and shaken for 1 h at room temperature. The resin was then filtered and washed with DCM (3×) and MeOH (3×), and dried under a vacuum for 16 h to afford globally (flask A + flask B) 5.47 g or resin **158**. Acidic mini cleavage (5% TFA/DCM, 10 min) of a sample of resin **158** and TLC analysis confirmed the complete deprotection of the amine. The loading yield, calculated by the mass increase, was 40%. It is noteworthy to mention that the loading yield could not be higher than 50% since 2 equivalents of trityl chloride resin were used for 1 equivalent of precursor **6b**. Then 25 bottom fritted reaction vessels of a 40 solid-phase reaction block of ACT LabTech manual synthesizer were loaded with 200 mg of resin **158**, which correspond to 0.102 mmol of compound **6b** (without the Fmoc protective group).

#### 4.2.18. Introduction of three levels of molecular diversity (library D)

The levels of molecular diversity were introduced by a similar procedure as described above in the preparation of libraries A, B and C. For the first level of molecular diversity, five stock solutions, each containing 2.5 equivalents (5x) of Fmoc-protected amino acid [Fmoc-*L*-Phe-OH (496 mg, 1.28 mmol), Fmoc-*L*-4-Pal-OH (Fmoc-*L*-4-pyridinealanine-OH) (497 mg, 1.28 mmol), Fmoc-*L*-Asp(O*t*Bu)-OH (527 mg, 1.28 mmol), Fmoc-*L*-Ser(Trt)-OH (729 mg, 1.28 mmol) or Fmoc-*L*-Tyr(2-Cl-Trt)-OH (870 mg, 1.28 mmol)], 2.5 equivalents (5x) of PyBrOP (595 mg, 1.28 mmol) and 2.5 equivalents (5x) of HOBt (175 mg, 1.28 mmol), were prepared in dry DMF (10 mL), and 5 equivalents (5x) of DIPEA (450 µL, 2.56 mmol) were added to each stock solution. To each of the 25 resins **158,** was added 2.0 mL of the appropriate stock solution. The mixtures were shaken under an argon atmosphere for 4 h at room temperature, then filtered, washed with DMF (2×), DCM (2×) and MeOH (3×), and dried under a vacuum to afford 5 groups of resins of general structure **159**. The completion of the coupling step was verified by acidic mini-cleavage and TLC analysis as described above. The cleavage of the Fmoc group of resins **159** was subsequently performed by a 1 h treatment with 2.0 mL of a solution of piperidine (20%) in DCM. The resins were then filtered, washed with DCM (3×), MeOH (3×) and DCM (1×), and dried under a vacuum to give 5 groups of resins of general structure **160**. The second level of molecular diversity was introduced using the same building blocks as for the first level except that Fmoc-*L*-4-Pal-OH was replaced by Fmoc-*L*-Ala-OH (398 mg, 1.28 mmol). The procedure described above for the introduction of the first level of molecular diversity was applied without modification. A TLC analysis after acidic mini-cleavage of a sampling of resins **161** confirmed that the introduction of the second level of molecular diversity was completed. Resins of general structure **161** were then treated with 2.0 mL of a solution of piperidine (20%) in DCM for 1 h at room temperature. The resins were filtered, washed with DCM (3×), MeOH (3×) and DCM (1×), and dried to afford resins of general structure **162**. The third level was introduced on resins **162** by coupling carboxylic acid building block **156**. A stock solution of **156** (1.60 g, 6.40 mmol), PyBrOP (2.98 g, 6.40 mmol) and HOBt (865 mg, 6.40 mmol) was prepared in dry DMF (50 mL) and DIPEA (2.23 mL, 12.8 mmol) was added. To each of the 25 resins **162** was added 2.0 mL of the stock solution. The mixtures were shaken under argon for 4 h at room temperature, then filtered, washed with MeOH (1×), DMF (2×), DCM (3×), MeOH (3×) and DCM (1×), and dried under a vacuum to afford resins of general structure **163**. The completion of the coupling reaction was verified by acidic mini-cleavage and TLC analysis.

#### 4.2.19. Removal of protective groups (library D)

Resins **163** that were prepared with Fmoc-*L*-Ser(Trt)-OH or Fmoc-*L*-Tyr(2-Cl-Trt)-OH building blocks were treated with 2.0 mL of a solution of TFA/TIS (triisopropylsilane)/DCM, 2:1:97 for 10 min at room temperature. The resins were filtered and washed with DCM (3×), MeOH (3×) and DCM (3×). This procedure was performed twice in order to completely remove the trityl or the 2-Cl-trityl protective groups. The deprotection step was completed when the solution of acidic mini-cleavage of a sampling of resins **163** (after step e) did not become yellow anymore. The THP protective group was next removed in mild acid condition. A solution of *p*-TSA (666 mg, 3.5 mmol) dissolved in *t*-BuOH (25 mL) and DCM (25 mL) was prepared and 2.0 mL of this solution was added to each of the 25 resins **163** (after step e, if necessary). The mixtures were shaken for 24 h at room temperature. They were then filtered, washed with DCM (3×), MeOH (3×) and DCM (1×), and dried under a vacuum to afford resins **164**.

#### 4.2.20. Generation of E_2_ derivatives 165 by nucleophilic cleavage

To each of the 25 resins **164** was added 2.0 mL of a solution of DEA (30%) in THF. The mixtures were shaken for 45 h at room temperature. Then 1.5 mL of a solution of DEA (30%) in THF was added to each of the 25 resins and the mixtures were shaken for an additional 20 h. The mixtures were then filtered and washed with THF (3×). The filtrates were collected in preweighed tubes and evaporated in a Speedvac apparatus. Each compound was dissolved in THF, evaporated twice and dried under a vacuum pump in order to obtain the DEA-free product. The phenol derivatives **165** were obtained in the form of a yellowish viscous oil. A random sampling was done and 2 compounds were analyzed by ^1^H NMR to verify their structure.

*16β-(N-[3′-(Acetylbutyl ester)]-phenylacetyl-L-tyrosine-L-phenylalanine-aminopropyl)-3,17β-dihydroxy-estra-1,3,5(10)-triene* (**165**: R_1_ = a, R_2_ = e). ^1^H-NMR (400 MHz, methanol-d_4_) δ 0.79 (s, 18-CH_3_), 0.85 to 2.35 (m, CH and CH_2_ of steroid skeleton and alkyl chain), 1.44 (s, (CH_3_)_3_CO), 2.78 (m, 6-CH_2_), 2.93 and 3.12 (2m, CHCH_2_Ph, CHCH_2_PhOH and CH_2_NH), 3.46 and 3.48 (2s, COCH_2_Ph), 3.51 (s, CH_2_COO*t*Bu), 3.75 (under THF peaks, 17α-CH), 4.52 (m, CHCH_2_Ph and CHCH_2_PhOH), 6.49 (d, *J* = 2.4 Hz, 4-CH), 6.55 (dd, *J_1_* = 8.4 Hz, *J_2_* = 2.6 Hz, 2-CH), 6.65 (d, *J* = 8.5 Hz, 3′-CH and 5′-CH of 4-hydroxyphenyl), 6.94 (d, *J* = 8.5 Hz, 2′-CH and 6′-CH of 4-hydroxyphenyl), 7.01 (d, *J* = 7.5 Hz, 1-CH), 7.14 (m, 9 × CH of phenyl).

*16β-(N-[3′-(Acetylbutyl ester)]-phenylacetyl-L-serine-L-aspartic acid-aminopropyl)-3,17β-dihydroxy-estra-1,3,5(10)-triene* (**165**: R_1_ = c, R_2_ = d). ^1^H-NMR (400 MHz, methanol-d_4_) δ 0.80 (s, 18-CH_3_), 0.85 to 2.35 (m, CH and CH_2_ of steroid skeleton and alkyl chain), 1.45 (s, (CH_3_)_3_CO), 2.78 (m, 6-CH_2_ and CH_2_COOH), 3.15 (m, CH_2_NH), 3.54 (s, COCH_2_Ph), 3.62 (s, CH_2_COO*t*Bu), 3.75 (under THF peaks, 17α-CH), 3.75 and 3.86 (under THF peaks and dd, *J_1_* = 10.6 Hz, *J_2_* = 5.5 Hz, CH_2_OH), 4.22 (t, *J* = 6.0 Hz, CHCH_2_OH), 4.75 (m, CHCH_2_COOH), 6.49 (d, *J* = 2.3 Hz, 4-CH), 6.55 (dd, *J_1_* = 8.4 Hz, *J_2_* = 2.6 Hz, 2-CH), 7.09 (d, *J* = 8.4 Hz, 1-CH), 7.24 (m, 4 × CH of phenyl).

#### 4.2.21. Generation of E_2_ derivatives (library D) by an acid treatment

In order to remove the *tert*-butyl ester protective group, each compound **165** was put in a tube and treated for 3 h at room temperature with 2.0 mL of HCl (4M in dioxane). The reaction mixtures were then evaporated to dryness under reduced pressure and dried overnight under a vacuum pump. The crude products were dissolved in MeOH and preadsorbed on C-18 silica gel. Purification by a C-18 silica gel column (Honeywell Burdick & Jackson, solid phase systems C18 columns, size 2000 mg/8 mL (product # 9009) distributed by VWR) afforded carboxylic acid, methyl ester or a mixture of carboxylic acid and methyl ester derivatives **166–190** (2–31 mg, 2–33% overall yield from **158**). LRMS and notes on ^1^H-NMR analysis of each library members are presented in Table 4. ^1^H-NMR of a random sampling of four members is described as examples.

**166** *(methyl ester)*. ^1^H-NMR (400 MHz, methanol-d_4_) δ 0.79 (s, 18-CH_3_), 0.95 to 2.35 (m, CH and CH_2_ of steroid skeleton and alkyl chain), 2.79 (m, 6-CH_2_), 2.90 and 3.10 (2m, 2 × CHCH_2_Ph and CH_2_NH), 3.45 and 3.46 (2s, COCH_2_Ph), 3.61 (s, CH_2_COOCH_3_), 3.67 (s, COOCH_3_), 3.71 (d, *J* = 9.8 Hz, 17α-CH), 4.53 (t, *J* = 7.3 Hz, COCHNH), 4.60 (m, COCHNH), 6.48 (d, *J* = 2.5 Hz, 4-CH), 6.55 (dd, *J_1_* = 8.4 Hz, *J_2_* = 2.5 Hz, 2-CH), 7.01 (d, *J* = 7.5 Hz, 1-CH), 7.05 to 7.30 (m, 14 × CH of phenyl).

**180** *(methyl ester)*. ^1^H-NMR (400 MHz, methanol-d_4_) δ 0.80 (s, 18-CH_3_), 1.00 to 2.35 (m, CH and CH_2_ of steroid skeleton and alkyl chain), 2.75 (m, 6-CH_2_), 2.80, 3.00 and 3.14 (3m, CHCH_2_COOH, CH_2_PhOH) and CH_2_NH), 3.54 (m, COCH_2_Ph), 3.63 and 3.66 (2s, CH_2_COOCH_3_), 3.68 (s, COOCH_3_), 3.71 (d, *J* = 9.7 Hz, 17α-CH), 4.48 (m, CHCH_2_PhOH), 4.64 (m, CHCH_2_COOH), 6.49 (d, *J* = 2.5 Hz, 4-CH), 6.55 (dd, *J_1_* = 8.3 Hz, *J_2_* = 2.6 Hz, 2-CH), 6.68 (m, 2 × CH of PhOH), 7.00 to 7.25 (m, 1-CH, 2 × CH of PhOH and 4 × CH of Ph).

**184** *(carboxylic acid)*. ^1^H-NMR (400 MHz, methanol-d_4_) δ 0.79 (s, 18-CH_3_), 0.95 to 2.35 (m, CH and CH_2_ of steroid skeleton and alkyl chain), 2.78 (m, 6-CH_2_), 3.20 (m, CH_2_NH), 3.60 and 3.63 (2s, COCH_2_Ph), 3.65 and 3.69 (2s, CH_2_COOH), 3.71 (d, *J* = 9.9 Hz, 17α-CH), 3.79 and 3.89 (2m, 2 × CH_2_OH), 4.39 and 4.45 (2m, 2 × CHCH_2_OH), 6.49 (d, *J* = 2.4 Hz, 4-CH), 6.55 (dd, *J_1_* = 8.4 Hz, *J_2_* = 2.5 Hz, 2-CH), 7.09 (d, *J* = 8.4 Hz, 1-CH), 7.24 (m, 4 × CH of Ph).

**185** *(mix of methyl ester and carboxylic acid)*. ^1^H-NMR (400 MHz, methanol-d_4_) δ 0.80 (s, 18-CH_3_), 0.90 to 2.35 (m, CH and CH_2_ of steroid skeleton and alkyl chain), 2.78 (m, 6-CH_2_), 2.90 and 3.05 (2m, CH_2_PhOH), 3.17 (m, CH_2_NH), 3.52 and 3.53 (2s, COCH_2_Ph), 3.58, 3.63 and 3.65 (3s, CH_2_COOX, × = H or CH_3_), 3.68 (s, COOCH_3_), 3.72 and 3.79 (2m, 17α-CH and CH_2_OH), 4.33 (m, CHCH_2_PhOH), 4.60 (m, CHCH_2_OH), 6.49 (s, 4-CH), 6.55 (dd, *J_1_* = 8.4 Hz, *J_2_* = 2.4 Hz, 2-CH), 6.68 (dd, *J_1_* = 8.4 Hz, *J_2_* = 1.8 Hz, 2 × CH of PhOH), 7.00 to 7.35 (m, 1-CH, 2 × CH of PhOH and 4 × CH of Ph).

### 4.3. Inhibition of 17β-HSD1 in homogenated cells (enzymatic assay)

The enzymatic assays on 17β-HSD1 were performed as previously described [15,41]. HEK-293 cells transfected with 17β-HSD1 cDNA fragment were briefly sonicated in 50 mM sodium phosphate buffer (pH 7.4) containing 20% glycerol and 1mM EDTA to obtain cellular fragmentation. The cytosol fraction containing the enzyme was isolated as the supernatant after centrifugation (100,000g, 5 min, 4 °C). The enzymatic reaction was performed at 37 °C for 2 h in 1 mL of a solution which included 870 µL of 50 mM sodium phosphate buffer (pH 7.4, 20% glycerol and 1 mM EDTA), 100 µL of 1 mM NADH in phosphate buffer, 10 µL of 10 µM [^14^C]-estrone in ethanol (54 mCi / mmol, American Radiolabeled Chemicals Inc., St-Louis, MO, USA), 10 µL of indicated inhibitor dissolved in ethanol and 10 µL of diluted enzymatic source in phosphate buffer. Each inhibitor was assessed in duplicate at a final concentration of 1µM. Radiolabeled steroids were then extracted twice from the reaction mixture by 1 mL of diethyl ether. The organic phases were pooled and evaporated to dryness with nitrogen. Residues were dissolved in 50 µL of DCM, applied on silica gel 60 F_254_ thin layer chromatography plates (EMD Chemicals Inc., Gibbstown, NJ, USA) and eluted with a mixture of toluene/acetone (4:1). Substrate ([^14^C]-E_1_) and metabolite ([^14^C]-E_2_) were identified by comparison with reference steroids and quantified using the Storm 860 system (Molecular Dynamics, Sunnyvale, CA, USA). The percentage of transformation of [^14^C]-E_1_ into [^14^C]-E_2_ was calculated as follows: % transformation = 100 × ([^14^C]-E_2_/([^14^C]-E_2_ + [^14^C]-E_1_)), and subsequently, % inhibition = 100 × [(% transformation without inhibitor - % transformation with inhibitor)/% transformation without inhibitor].

## Figures and Tables

*Reagents and conditions*: (a) H_2_, 5% Pd/C, EtOAc, MeOH, rt, 2 h (for **4a**); (b) H_2_, 5% Pd/C, MeOH, rt, 3 h (for **4b**); (c) TBAF, THF, 0 °C, 30 min (for **4a**); (d) Fmoc-OSu, NaHCO_3_, THF/H_2_O (3:1, v/v), 0 °C, 1 h (for **5a** and **5b**); (e) NH_2_SO_2_Cl, 2,6-di-*t*-butyl-4-methylpyridine, DCM, rt, 16–24 h (**6a**: 82% for 4 steps; **6b**: 52% for 3 steps).

**Scheme 2:**
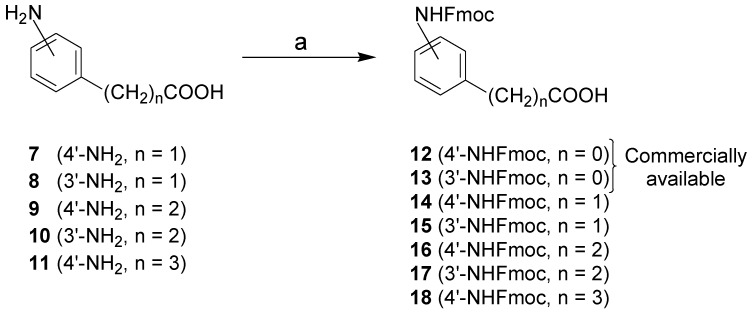
Synthesis of N-Fmoc protected carboxylic acid building blocks **14–18**.

*Reagents and conditions*: (a) Fmoc-OSu, NaHCO_3_, THF/H_2_O (5:1, v/v), rt, 16 h (51–93%).

**Scheme 3:**
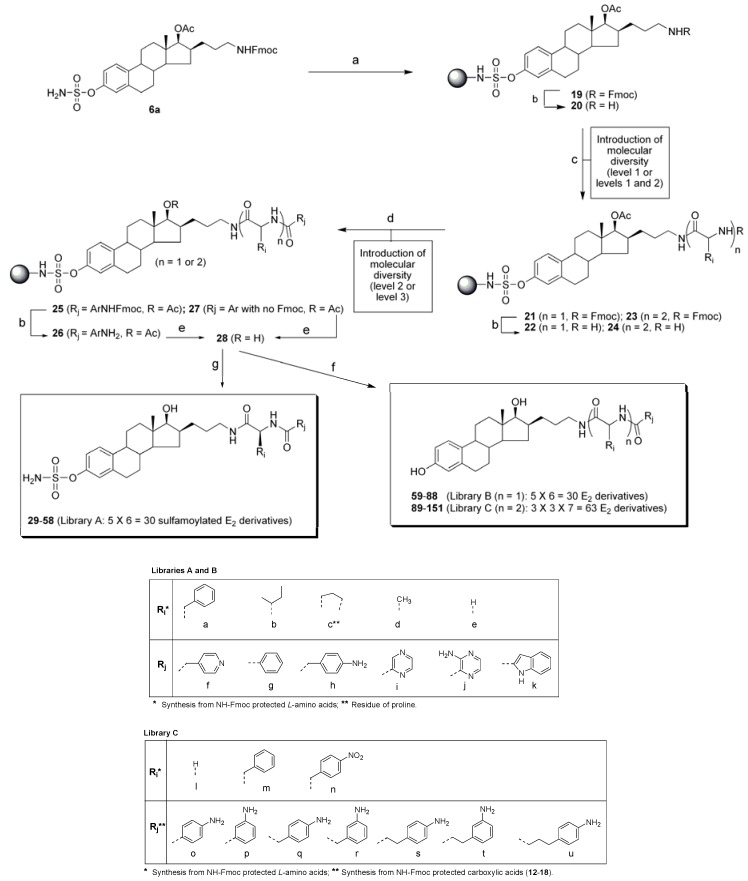
Preparation of libraries A, B and C.

*Reagents and conditions*: (a) Trityl chloride resin, DIPEA, DCM, rt, 12–16 h (libraries A and B: loading of 70%, library C: loading of 42%); (b) 20% piperidine/DCM (v/v), rt, 1 h; (c) PyBOP, HOBt, R_i_CH(NHFmoc)COOH, DIPEA, DMF, rt, 2–3 h; (d) PyBrOP, HOBt, R_j_COOH or **14 **(libraries A and B), **12–18 **(library C), DIPEA, DMF, rt, 2–3 h; (e) 1M MeONa/MeOH : THF (25/75, v/v) rt, 48–100 h; (f) 30% DEA/THF v/v, rt, 24–66 h (library B: **59–88**, 10–63% for 6 or 7 solid-phase steps, library C: **89–151**, 6–41% for 9 solid-phase steps); (g) 30% HFIP/DCM, rt, 6 h (library A: **29–58**, 35–53% for 6 or 7 solid-phase steps).

**Scheme 4:**
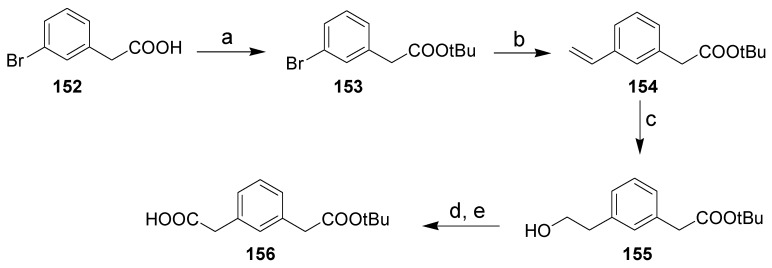
Synthesis of carboxylic acid building block **156**.

*Reagents and conditions*: (a) BOC_2_O, DMAP, *t*-BuOH, rt, 24 h (94%); (b) tributyl(vinyl)tin, Pd_2_(dba)_3_, P(*t*-Bu)_3_ (10% w/v in hexanes), toluene, rt, 16 h (88%); (c) *i*. BH_3_.THF, THF, 0 °C, 3 h; *ii*. NaOAc (4M), H_2_O_2_ (30% w/v), 0 °C, 2 h (45%); (d) Dess-Martin periodinane, DCM, rt, 1 h (78%); (e) NaClO_2_, NaH_2_PO_4_, H_2_O, 2-methyl-2-butene, *t*-BuOH, rt, 15 min (88%).

**Scheme 5:**
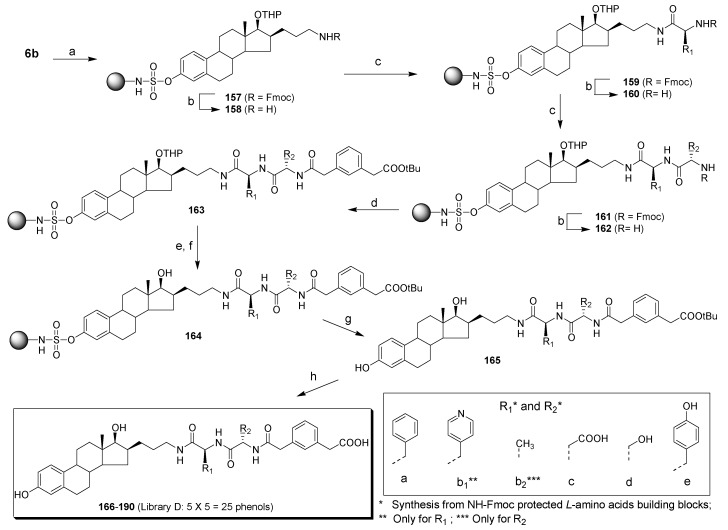
Preparation of library D.

*Reagents and conditions*: (a) Trityl chloride resin, DIPEA, DCM, rt, 16 h (loading: 40%); (b) 20% piperidine/DCM (v/v), rt, 1 h; (c) PyBrOP, HOBt, R_1_CH(NHFmoc)COOH, DIPEA, DMF, rt, 4 h; (d) PyBrOP, HOBt, **156**, DIPEA, DMF, rt, 4 h; (e) TFA/TIS/DCM (2:1:97), rt, 2 × 10 min; (f) *p*-TSA, *t*-BuOH, DCM, rt, 24 h; (g) 30% DEA/THF v/v, rt, 65 h; (g) HCl (4M in dioxane), rt, 3 h (2–33% for 8 solid-phase steps and 1 solution-phase step).

**Table 1:**
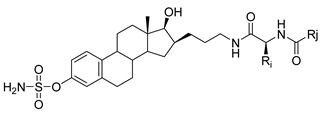
Characterization of members from library A (sulfamoylated E_2_ derivatives **29–58**).

Compounds	R_i_*^a^*	R_j_*^a^*	LRMS [M + H]^+^ (m/z)		Overall yield	HPLC purity
			calculated	measured	(%)*^b^*	(%)
**29**	a	f			50	
**30**	a	g	660.3	660.4	45	89
**31**	a	h			46	
**32**	a	i			53	
**33**	a	j			46	
**34**	a	k			45	
**35**	b	f			45	
**36**	b	g			42	
**37**	b	h			39	
**38**	b	i	628.3	628.4	50	90
**39**	b	j			47	
**40**	b	k			39	
**41**	c	f	625.3	625.4	47	89
**42**	c	g			53	
**43**	c	h			47	
**44**	c	i			49	
**45**	c	j			51	
**46**	c	k			53	
**47**	d	f			51	
**48**	d	g			49	
**49**	d	h	613.3	613.3	38	91
**50**	d	i			50	
**51**	d	j	601.3	601.3	47	90
**52**	d	k			43	
**53**	e	f			49	
**54**	e	g			42	
**55**	e	h			42	
**56**	e	i			42	
**57**	e	j			35	
**58**	e	k	609.3	609.2	41	89

*^a^* See Scheme 3 for the chemical structure of R building blocks; *^b^* Crude overall yields calculated for the solid-phase sequence of 6 or 7 steps.

**Table 2:**
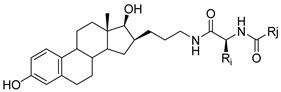
Characterization of members from library B (E_2_ derivatives **59–88**).

Compounds	R_i_*^a^*	R_j_*^b^*	LRMS [M + H]^+^ (m/z)		Overall yield	HPLC purity
			calculated	measured	(%)*^b^*	(%)
**59**	a	f			50	
**60**	a	g			35	
**61**	a	h	610.4	610.4	26	85
**62**	a	i			63	
**63**	a	j			35	
**64**	a	k			39	
**65**	b	f			23	
**66**	b	g			26	
**67**	b	h			42	
**68**	b	i			18	
**69**	b	j	564.4	564.5	22	95
**70**	b	k			15	
**71**	c	f			41	
**72**	c	g			37	
**73**	c	h			46	
**74**	c	i	533.3	533.4	43	91
**75**	c	j			12	
**76**	c	k			47	
**77**	d	f			29	
**78**	d	g	505.3	505.5	21	90
**79**	d	h			14	
**80**	d	i			13	
**81**	d	j			16	
**82**	d	k	544.3	544.5	13	89
**83**	e	f	506.3	506.5	28	88
**84**	e	g			22	
**85**	e	h			12	
**86**	e	i			13	
**87**	e	j			10	
**88**	e	k			14	

*^a^* See Scheme 3 for the chemical structure of R building blocks; *^b^* Crude overall yields calculated for the solid-phase sequence of 6 or 7 steps.

**Table 3A:**
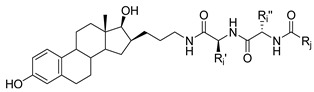
Characterization of members from library C (E_2_ derivatives **89–123**).

Compounds	R_i_′	R_i_″	R_j_*^a^*	LRMS [M + H]^+^ (m/z)		Overall yield
				calculated	measured	(%)*^b^*
**89**	l	L	o	563.3	563.3	27
**90**	l	L	p			28
**91**	l	L	q			22
**92**	l	L	r			25
**93**	l	L	s			25
**94**	l	L	t	589.3*^c^*	589.5*^c^*	26
**95**	l	L	u			28
**96**	l	M	o			36
**97**	l	M	p			38
**98**	l	M	q			30
**99**	l	M	r	665.4*^c^*	665.5*^c^*	28
**100**	l	M	s			33
**101**	l	M	t			30
**102**	l	M	u			30
**103**	l	N	o	698.3	698.2	18
**104**	l	N	p			30
**105**	l	N	q			27
**106**	l	n	r			26
**107**	l	n	s			30
**108**	l	n	t			28
**109**	l	n	u	740.4	740.5	32
**110**	m	l	o			34
**111**	m	l	p	653.4	653.2	40
**112**	m	l	q			23
**113**	m	l	r			32
**114**	m	l	s	681.4	681.5	35
**115**	m	l	t			35
**116**	m	l	u			37
**117**	m	m	o			39
**118**	m	m	p	743.4	743.2	41
**119**	m	m	q			31
**120**	m	m	r			35
**121**	m	m	s	771.4	771.6	35
**122**	m	m	t			38
**123**	m	m	u			38

^a^ See Scheme 3 for the chemical structure of R building blocks. *^b^* Crude overall yields calculated for the solid-phase sequence of 9 steps. *^c^* [M-H]^-^.

**Table 3B:**
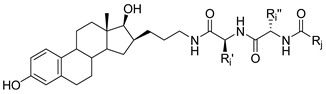
Characterization of members from library C (E_2_ derivatives **124–151**).

Compounds	R_i_′	R_i_″	R_j_*^a^*	LRMS [M + H]^+^ (m/z)		Overall yield
				calculated	measured	(%)*^b^*
**124**	m	n	o			38
**125**	m	n	p			31
**126**	m	n	q	802.4	802.4	32
**127**	m	n	r			33
**128**	m	n	s			34
**129**	m	n	t			34
**130**	m	n	u			37
**131**	n	l	o			31
**132**	n	l	p			40
**133**	n	l	q	712.4	712.5	26
**134**	n	l	r			28
**135**	n	l	s			30
**136**	n	l	t			28
**137**	n	l	u			34
**138**	n	m	o			34
**139**	n	m	p			36
**140**	n	m	q			29
**141**	n	m	r			32
**142**	n	m	s			28
**143**	n	m	t	816.4	816.4	33
**144**	n	m	u	828.4*^c^*	828.3*^c^*	36
**145**	n	n	o			33
**146**	n	n	p			6
**147**	n	n	q			26
**148**	n	n	r	847.4	847.3	30
**149**	n	n	s			28
**150**	n	n	t			28
**151**	n	n	u			31

*^a^* See Scheme 3 for the chemical structure of R building blocks.*^b^* Crude overall yields calculated for the solid-phase sequence (9 steps). *^c^* [M-H]^-^.

**Table 4:**
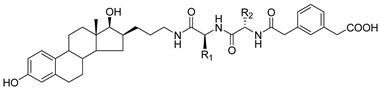
Characterization of members from library D (E_2_ derivatives **166–190**).

Compounds	R_1_*^a^*	R_2_*^a^*	LRMS [M + H]^+^ (m/z)	Overall yield	Note on ^1^H-NMR analysis	
			calculated	measured	(%)*^b^*	
**166**	**a**	**a**	**800.4**	**814.3***^c^*	**18**	**ester**
**167**	a	b_2_	722.4*^d^*	722.4*^d^*	26	mix of ester and acid
**168**	a	c	766.4*^d^*	766.7*^d^*	4	acid
**169**	a	d	738.4*^d^*	738.4*^d^* + 752.4*^c^*	7	mix of ester and acid
**170**	a	e	816.4	816.3	17	acid
**171**	b_1_	a	801.4	801.3 + 815.4*^c^*	29	mix of ester and acid
**172**	b_1_	b_2_	723.4*^d^*	723.5*^d^* + 737.6*^c^*	26	mix of ester + acid + impurities
**173**	b_1_	c	767.4	781.5*^c^*	22	ester
**174**	b_1_	d	741.4	not found	0	impurities
**175**	b_1_	e	817.4	831.4*^c^*	16	ester
**176**	c	a	766.4*^d^*	780.5*^c^*	26	ester
**177**	c	b_2_	690.4*^d^*	690.4*^d^*	2	mix of acid + impurities
**178**	c	c	734.3*^d^*	734.4*^d^*	28	acid
**179**	c	d	708.3	708.1 + 722.1*^c^*	10	mix of ester + acid + impurities
**180**	c	e	782.4*^d^*	796.5*^c^*	20	ester
**181**	d	a	740.4	not found	0	impurities
**182**	d	b_2_	662.4*^d^*	676.4*^c^*	4	ester
**183**	d	c	706.3*^d^*	706.3*^d^*	19	mix of acid + impurities
**184**	d	d	678.3*^d^*	678.3*^d^*	3	acid
**185**	d	e	754.4*^d^*	754.4*^d^* + 768.4*^c^*	5	mix of ester and acid
**186**	e	a	816.4	816.3 + 830.3*^c^*	21	mix of ester and acid
**187**	e	b_2_	738.4*^d^*	738.3*^d^* + 752.4*^c^*	33	mix of ester and acid
**188**	e	c	782.4*^d^*	782.5*^d^* + 796.4*^c^*	31	mix of ester and acid
**189**	e	d	754.4*^d^*	754.3*^d^*	7	acid
**190**	e	e	830.4*^d^*	830.4*^d^*	18	acid

*^a^* See Scheme 5 for the chemical structure of R building blocks. *^b^* Overall yields calculated for the solid phase sequence of nine steps after C-18 silica gel purification. *^c^* M+14; *^d^* LRMS [M-H]^-^.
